# Engineered Cell Membrane-Camouflaged Nanomaterials for Biomedical Applications

**DOI:** 10.3390/nano14050413

**Published:** 2024-02-23

**Authors:** Xiyuan Guan, Simin Xing, Yang Liu

**Affiliations:** Department of Chemistry, Beijing Key Laboratory for Analytical Methods and Instrumentation, Kay Lab of Bioorganic Phosphorus Chemistry and Chemical Biology of Ministry of Education, Tsinghua University, Beijing 100084, China; gqy23@mails.tsinghua.edu.cn (X.G.); xingsm21@mails.tsinghua.edu.cn (S.X.)

**Keywords:** engineered cell membrane, cell membrane-camouflaged nanomaterials, long circulation time, active targeting, biomedical applications

## Abstract

Recent strides in nanomaterials science have paved the way for the creation of reliable, effective, highly accurate, and user-friendly biomedical systems. Pioneering the integration of natural cell membranes into sophisticated nanocarrier architectures, cell membrane camouflage has emerged as a transformative approach for regulated drug delivery, offering the benefits of minimal immunogenicity coupled with active targeting capabilities. Nevertheless, the utility of nanomaterials with such camouflage is curtailed by challenges like suboptimal targeting precision and lackluster therapeutic efficacy. Tailored cell membrane engineering stands at the forefront of biomedicine, equipping nanoplatforms with the capacity to conduct more complex operations. This review commences with an examination of prevailing methodologies in cell membrane engineering, spotlighting strategies such as direct chemical modification, lipid insertion, membrane hybridization, metabolic glycan labeling, and genetic engineering. Following this, an evaluation of the unique attributes of various nanomaterials is presented, delivering an in-depth scrutiny of the substantial advancements and applications driven by cutting-edge engineered cell membrane camouflage. The discourse culminates by recapitulating the salient influence of engineered cell membrane camouflage within nanomaterial applications and prognosticates its seminal role in transformative healthcare technologies. It is envisaged that the insights offered herein will catalyze novel avenues for the innovation and refinement of engineered cell membrane camouflaged nanotechnologies.

## 1. Introduction

With the rapid development of nanomedicine, nanoparticles have burgeoned into a preeminent subject of inquiry, chiefly buoyed by their envisaged role in enhancing the diagnostics, therapeutics, and prophylaxis of various afflictions [1,2]. Encapsulation of therapeutic agents within nanoparticles has been established as a noteworthy strategy to mitigate systemic toxicity and bolster the durability of drugs [3,4]. To further prolong the circulation time and targeting ability of nanoparticles in the blood, different methods have been explored [5]. A case in point involves the initial benchmark of employing polyethylene glycol (PEG) for the creation of stealth nanoparticle coatings, although it was later discerned that PEG modification could inadvertently trigger an immunogenic response, culminating in the hastened eradication of the nanoparticles [6]. In the active targeting strategy, advancements have focused on enhancing the site-specific aggregation by chemically or physically connecting the targeted ligands to the nanoparticle surface [7,8]. This leverages the specificity intrinsic to antibody–antigen recognition or ligand–receptor interactions to direct therapeutic payloads with precision [9]. However, concerns have been raised regarding the fidelity of physical adsorption due to its propensity for instability and lack of dependability, while chemical modifications, although more stable, present as convoluted and laborious, thus impeding large-scale synthesis.

In a pioneering study conducted in 2011, Zhang et al. utilized membranes derived from red blood cells (RBCs) to engineer a deceptive veneer for nanoparticles, significantly amplifying the circulatory half-life of nanoparticles [10]. This is because the presence of an RBC membrane cloak diminishes the macrophage uptake. Presently, various types of cells are being harnessed in cell membrane camouflaging nanotechnology, and the nanoparticles can be organic/inorganic nanoparticles with multifarious functions [11,12]. The praise for this nanotechnological platform stems from its ability to mimic complex cellular functions, forming a communication between nanoparticles and the internal microenvironment [13]. For example, by inheriting self-recognizing epitopes from their cellular progenitors, these nanoparticles deftly navigate the immune surveillance [14]. Furthermore, certain progenitor cells have multiple affinity ligands that possess efficient homologous targeting, bestowing upon these chimeric particles an innate propensity for homing in on specific tissues or pathologies [15].

While the cell membrane camouflage grants these nanoparticles an exceptional capacity for evasion, additional target selectivity may further limit off-target side effects and bolster the efficacy of the clinical intervention [16]. With the deployment of cell membrane-camouflaged nanomaterials in complex biological systems, the clamor for enhanced multifunctionality in such systems becomes ever more acute [17]. Cell membrane engineering emerges as a salient strategy, which aims to enhance or endow cell membranes with new functions by modifying and manipulating their composition and structure [18]. This innovative form of engineering involves inserting or modifying membrane proteins, lipid compositions, or other membrane-related molecules, which can be used to improve drug delivery, potentiate cellular therapies, or develop the next generation of biosensors. Additional target modifications may further limit off-target side effects and improve therapeutic efficacy: for instance, a hybrid membrane combining ID8 ovarian cancer cell membranes with RBCs demonstrates a pronounced affinity for homing characteristics towards ID8 cells [19]. Similarly, the engineering of hybrid membranes, cloaking nanoliposomes with macrophage and RBC membranes, can actively direct them to inflammatory loci [20]. Moreover, the fusion of platelet and RBC membranes has shown a chemotaxis effect on atherosclerosis [21]. Augmenting the inherent characteristics of cell membranes through such engineering not only extends the horizons of biomimetic nanoparticle application but also signals a paradigm shift in how nanomedicine can capitalize on nature’s designs to foster innovations that transcend the limitations of the biological entities from which they draw inspiration.

**Figure 1:**
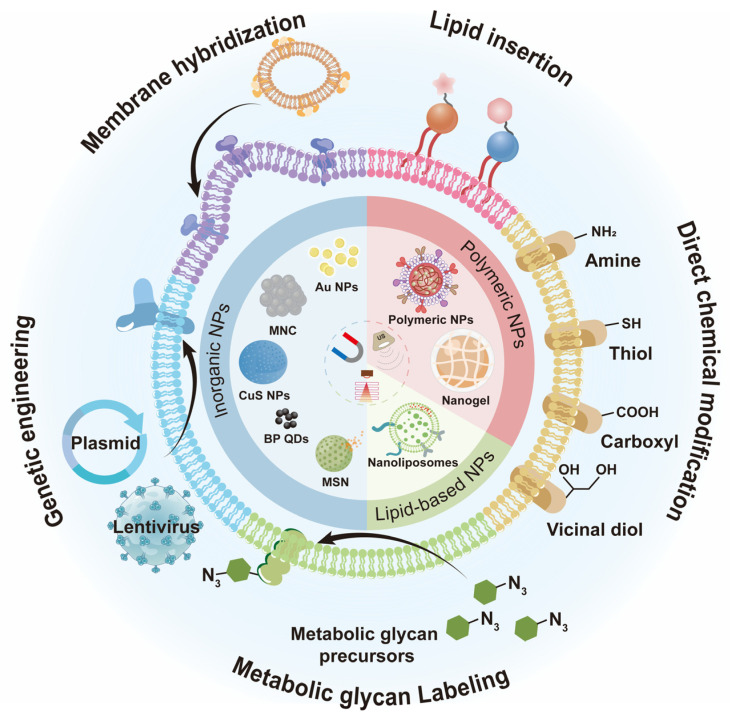
Schematic of engineered cell membrane-camouflaged nanomaterials. The lentivirus in Figure 1 was drawn by the Figdraw 2.0 (https://www.figdraw.com, accessed on 25 December 2023).

At the crossroads of cell membrane engineering, this review undertakes a succinct synthesis of the prevalent techniques in cell membrane engineering. From this vantage point, we summarize the research progress of the application of engineered cell membrane camouflage strategies, delineating them by material classification, as shown in Figure 1. This approach facilitates a more cohesive understanding of the field’s current developments. Concluding our discourse, we also emphasized some challenges and opportunities that shape the evolution of cell membrane camouflage strategies, hoping to encourage further detailed investigation.

## 2. Strategies for Engineered Cell Membranes

With the in-depth exploration and application of cell membrane camouflage nanotechnology, especially in the field of nanomedicine, the demand for further expanding the specific functions of natural cell membranes is constantly increasing. As previously delineated, the potential application of this nanotechnology in clinical practice is severely limited due to inherent biological function limitations, manifesting as constraints in specific targeting and stimulus responsiveness [22]. While maintaining their biomimetic benefits, engineered cell membranes can be endowed with additional functionalities in certain fields [23]. Consequently, the pursuit of concocting engineered cell membranes with attributes that transcend those of their natural counterparts is now captivating the scientific community, energizing a diverse spectrum of innovative methodologies (Table 1) [24].

### 2.1. Lipid Insertion

**Figure 2:**
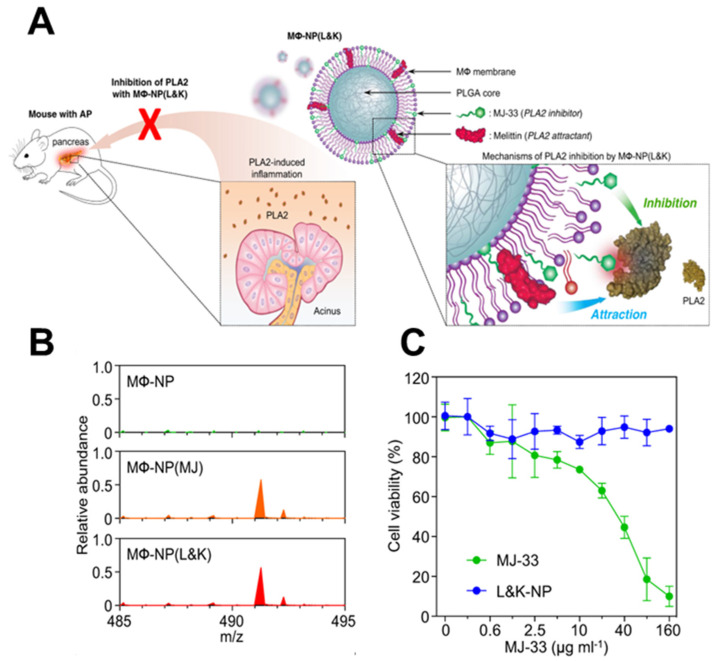
Construction of a nano platform for insertion of melittin into cell membranes. (**A**) The membrane of MΦ can be used as a coating material to divert PLA2 from its intended target cell. (**B**) A molecular ion peak was identified at *m*/*z* = 491.3, which corresponds to the theoretical molecular weight of MJ−33. This indicates that MJ−33 was successfully loaded into the nanoparticle formulations. (**C**) While free MJ−33 was found to be toxic to macrophages, MΦ−NP(L&K) containing the same amount of MJ−33 was non−toxic. (MΦ: macrophages; AP: acute pancreatitis; PLA2: phospholipase A2; PLGA: polylactic acid−co−glycolic acid; MJ−33: PLA2 inhibitor). Reprinted from [29] with permission from Springer Nature, open access, copyright 2021.

Lipid insertion involves modifying the cell membrane by attaching a hydrophobic anchor to the functional ligand. Given the phospholipid bilayer structure of the cytoskeleton, the hydrophobic anchor is driven by the hydrophobic effect and inserts spontaneously, thereby binding functional ligands [25]. Compared to chemical modification, lipid insertion maintains the original properties of the cell membrane to a greater extent while safeguarding its integrity. The category of hydrophobic anchors can be separated into single and multiple anchor formats depending on the number of drain anchors [26]. Double anchors provide a more stable structure after insertion than single anchors. 1,2-distearoyl-snglycero-3-phosphoethanolamine (DSPE) is frequently utilized as a double-anchored hydrophobic group, while polyethylene glycol (PEG) spacers are commonly incorporated during research to retain the ligand’s biological activity. Shi et al. inserted a single anchor DNA initiator onto the natural killer (NK) cell membrane [27]. They constructed a supramolecular aptamer-based polyvalent antibody mimic (PAM) by in situ hybridization of DNA scaffolds with multiple aptamers. The PAM exhibits efficient targeting, adhesion, and killing effects on tumor cells. Similarly, Zhang et al. employed melittin to form a nano platform (M Φ- NP (L&K) for treating acute pancreatitis by inserting it into the macroscopic membrane, avoiding the disadvantage of severe hemolysis caused by intravenous administration of melittin (Figure 2) [28,29]. In a separate study, folate–linker–lipid conjugates (1,2-distearoyl-sn-glycerol-3 (FITC-PEG-lipid) phosphoethanolamine-N-[folate (polyethylene glycol)-2000]) were synthesized, and then ligand–lipid conjugates were integrated into the RBC membrane [30]. This functionalization enables the RBC membrane to target model tumors actively.

In numerous cases, the insertion of lipids into the cell membrane lacks specificity, resulting in haphazard interaction between lipids and the cell surface. This indiscriminate interaction can potentially undermine the cell’s vitality and functionality. Additionally, due to membrane flow and endocytosis, functional materials introduced into the cell membrane via lipid insertion are susceptible to loss, thereby restricting their wider usage [31].

### 2.2. Membrane Hybridization

**Figure 3:**
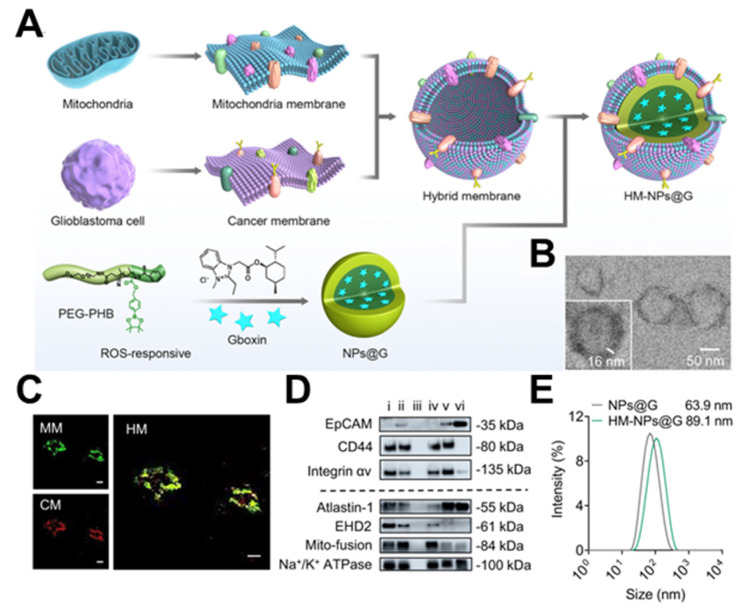
Construction of HM−NPs@G. (**A**) Methods based on cancer cell–mitochondria hybrid membrane−camouflaged Gboxin−encapsulated ROS−responsive polymeric nanoparticles. (**B**) TEM images showing the core–shell structure of the developed HM−NPs@G. Scale bar  =  50 nm. (**C**) CLSM images of the successful fusion of CM and MM. Scale bar = 20 μm. (**D**) Western blotting analysis of cancer membrane and mitochondria membrane special targeting−related key proteins. (**E**) Size distribution of NPs@G (without membrane coating) and HM−NPs@G. (TEM: transmission electron microscope; PEG−PHB: poly(ethylene glycol)-poly(4-(4,4,5,5-Tetramethyltetramethyl-1,3,2-dioxaborolan-2-yl)benzyl acrylate); MM: mitochondria membrane; CM: cancer membrane; HM: hybrid membrane; EpCAM: epithelial cell adhesion molecule). Reprinted from [33] with permission from Springer Nature, open access, copyright 2023.

Each source of membranes has both advantages and disadvantages. Membrane hybridization is a technique that merges two or more membranes from different sources, depending on the fluidity of the phospholipid layer [32]. Ultrasonic and extrusion methods can be used to obtain hybrid membranes, which can combine a variety of biological functions and allow more complex functions [33]. Tumor cells are effortlessly expandable in vitro, and it is easy to access their cell membrane. The autoimmune evasion property of the tumor cell membrane can prolong the nanoparticles’ circulation period. Additionally, tumor cell membranes contain self-recognizing molecules like N-cadherin and Thompson–Friedenreich antigen, which promote effective homologous targeting [34]. Compared to the active tumor targeting method based on one single ligand receptor, this approach is more effective and accurate. Additionally, the tumor cell membrane performs other functions; for instance, the surface receptors of the glioblastoma (GBM) membrane can interact with endothelial cell adhesion molecules, facilitating the crossing of the blood–brain barrier (BBB). Chi et al. coated nanomedicines with the cell membranes of brain metastatic breast cancer MCF-7 and GBM U87-MG, demonstrating their ability to cross the BBB and target homologous tumors [35]. Similarly, Yan et al. developed a reactive oxygen species (ROS)-responsive nanoparticle (HM-NPs@G) masked by a hybrid membrane consisting of cancer cells and mitochondria to target GBM mitochondria with the oxidative phosphorylation inhibitor Gboxin, which effectively prolonged the circulation time, facilitated the permeability of the BBB, and improved its accumulation at the GBM site (Figure 3) [33].

The combination of various cell membranes and immune cell membranes allows the biomimetic membrane to attain functions of inflammation targeting and immunotherapy, which hold great potential. For instance, Zhou et al. utilized the inflammatory homing properties of macrophage membranes to fabricate nanoparticles disguised by a hybrid membrane of platelets and macrophages (RAW 264.7 cells) to effectively dispatch small interfering RNA sirsav1 to target inflammatory heart cells [36]. For another example, dendritic cells (DCs) can present tumor antigens through their major histocompatibility complexes, activate antigen-specific T cells, and destroy homologous tumor cells [37]. By fusing the membranes of specific tumor cells and DCs, researchers obtained a mixed membrane that effectively expresses tumor-specific antigens and immune-costimulatory molecules. This substance possesses tumor self-targeting properties and promotes the maturation of DCs and cytotoxic T lymphocytes (CTLs) in the lymph nodes. For example, Xu et al. coated semiconductor polymer nanoparticles with the hybrid membrane of 4T1 tumor cells and DCs [38]. After undergoing near-infraredⅡ (NIR-II) irradiation, the photothermal effect facilitated by semiconductor polymer collaborates with immunotherapy stimulated by engineered membranes, thwarts tumor expansion, and impedes metastasis.

The potential of membrane hybridization technology to amalgamate functional proteins derived from diverse cellular membranes is tempered by the challenges associated with identifying a compatible fusion strategy for distinct membrane types [39]. Such impediments could precipitate the incorporation of extraneous molecules, with consequent deleterious responses.

### 2.3. Direct Chemical Modification

Direct chemical modification occurs through the formation of covalent bonds between the hydroxyl residues of polysaccharides or the amino groups (-NH_2_), carboxyl groups (-COOH), and sulfhydryl groups (-SH) of proteins on the cell membrane and functional molecules. The primary advantage of the direct chemical modification approach is that synthetic molecules can be covalently modified in a relatively stable manner without the requirement for prior cell treatment [32,40].

**Figure 4:**
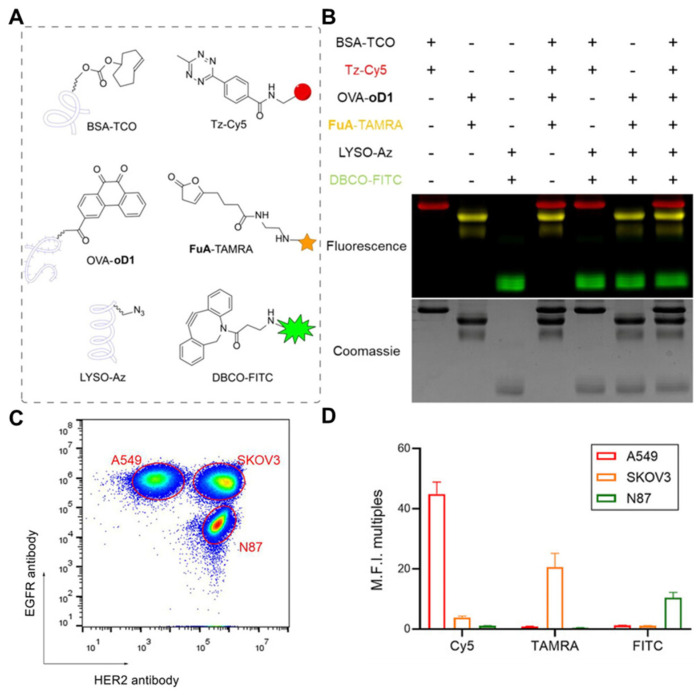
Development of direct chemical modification. (**A**) BSA−TCO, OVA−oD1, and LYSO−Az were reacted with different fluorophore−modified bioorthogonal compounds. (**B**) SDS−PAGE analysis result of orthogonal labeling of three different proteins based on DFC, SPAAC, and IEDDA reactions. (**C**) Distinguishing three different cell groups using EGFR and HER2 antibodies. (**D**) Fluorescence signal analysis indicates that A549 (TCO) can only be modified by the IEDDA reaction. (BSA: bovine serum albumin; TCO: trans−cyclooctene; Tz: Phenyl−methyl−tetrazine; Cy5: fluorescent dyes; OVA: ovalbumin; oD1: o−dione; SDS−PAGE: sodium dodecyl sulfate–polyacrylamide gel electrophoresis; FuA: furan−2 (3H)−one substrate; TAMRA: Carboxytetramethylrhodamine; LYSO: lysozyme; Az: azide; DBCO: Dibenzocyclooctyne; FITC: Fluorescein Isothiocyanate; DFC: o−dione and furan−2 (3H)−one cycloaddition; SPAAC: strain−promoted azide–alkyne click reaction; IEDDA: inverse−electron−demand Diels–Alder reaction). Reprinted from [43] with permission from Springer Nature, copyright 2023.

There are numerous -NH_2_ present at the N-terminus and lysine residues of protein on the cell membrane, which can be chemically modified by acylation reaction [41]. For example, Cai et al. modified human umbilical vein endothelial cells (HUVECs) and human skin fibroblasts (HSFs) with succinimide ester-methoxy polyethylene glycol (NHS-mPEG), which enhanced the migration ability of cells [42]. Xi et al. labeled the amino groups of SKOV3 cell membrane proteins with N-hydroxysuccinimide (NHS) ester of furan-2(3H)-one substrate (FuA) (FuA-NHS), which quickly binds to o-dione-carboxytetramethyl-rhodamine and exhibits fluorescence (Figure 4) [43]. Moreover, there is a significant presence of carboxyl groups (-COOH) at the C-terminus and aspartic acid and glutamic acid residues of protein on the cell membrane. The amidation reaction mediated by 1-ethyl-3-(3-dimethylaminopropyl) (EDC) is generally utilized [44]. This technique has shown long-term efficacy and safety in coupling the antigen with cells when used in experimental models of autoimmune diseases, transplantation tolerance, and allergic diseases. Additionally, maleimide derivatives can form stable thioether bonds with the sulfhydryl groups on the protein via the Michael addition reaction [45]. Based on this, Wang et al. developed PEGylated solid lipid nanoparticles that were functionalized with maleimide groups [46]. The functionalization enhanced the adsorption onto RBCs, suggesting the potential of these nanoparticles for targeted drug delivery.

Sialic acid (SA) is abundant on the cell membrane containing numerous o-diol groups, which can uniquely form dynamic covalent bonds with phenylboronic acid derivatives. For example, Tao et al. used the fluorescent polymer 1,4-dihydropyridine (1,4-DHP) to image the cell membrane by interacting phenylboronic acid (PBA) with sialic acid [47].

Although direct chemical modification stands as a pragmatic approach to functionalizing cell membranes, it is challenging to control the modification site on the membrane. This lack of precision at the molecular level means that the ensuing chemical reactions may occur indiscriminately. Given the functional specificity of proteins and polysaccharides integral to the cell membrane architecture, such nonspecific modifications harbor the potential risk of impairing their intrinsic functionality, thus undercutting the biological efficacy of the modified membranes [48].

### 2.4. Metabolic Glycan Labeling

**Figure 5:**
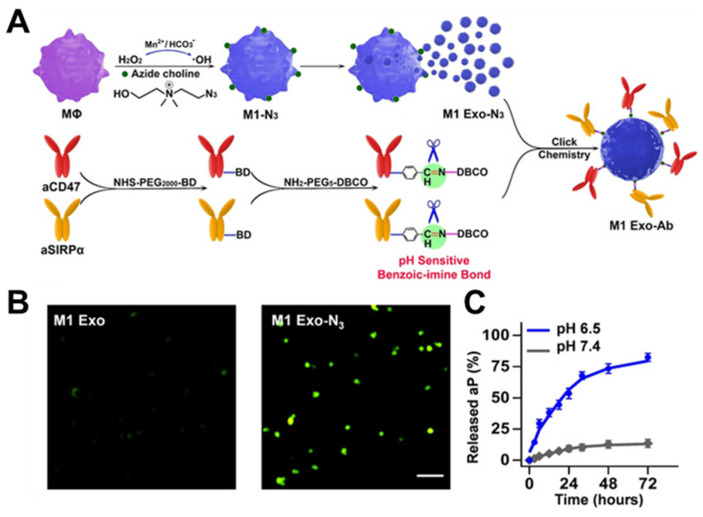
Development of metabolic glycan labeling. (**A**) The synergism of M1 Exo-Ab. (**B**) Fluorescence imaging of pristine M1 Exo and M1 Exo-N_3_ after incubation with DBCO-Cy5 confirmed M1 Exo carried azide groups on their surface. (**C**) Release profiles of total Ab from M1 Exo-Ab at different pHs (6.5 and 7.4) demonstrating the selective cleavage of the benzoic-imine bond. (M1 Exo-Ab: exosome nanobioconjugates; MΦ: macrophages; aCD47: anti-CD47 antibody; SIRPα: signal regulatory proteins; Ab: antibody). Reprinted from [50] with permission from Wiley, copyright 2019.

Since the initial introduction of exogenous glycans into the glycocalyx of the cell membrane by Bertozzi and her colleagues, the approaches to modifying the cell membrane with metabolic glycans have progressively advanced [49]. Their seminal development has become a powerful tool in the field of chemical biology, enabling precise modifications of the cell surface with minimal disruption to cell function. In this procedure, non-natural glycan precursors containing functional groups, such as azides, alkynes, mercaptans, and alkenes, are metabolically incorporated into the glycan residues. Functional molecules can subsequently be linked via bioorthogonal reactions. Bioorthogonal reactions can be carried out under physiological conditions, in which the natural structure and function of cell membranes are preserved (Figure 5) [50]. Compared to direct chemical modification, this method can be implemented in a gentle physiological setting without compromising the biochemical processes and significantly enhances the density of active sites on the cell surface. Because of substantial modifications of syringic acid (SA) residues on the tumor cells, SA metabolic precursors with azido groups, N-azidoacetylmannosamine tetraacetate (Ac_4_ManNAz), have been commonly employed [51]. These membranes are then coated onto the nanoparticle surface using electrostatic interaction, which facilitates click chemistry reactions for functionalization [52].

The strategic employment of metabolic glycan precursors to engineer cell membrane modifications in vitro presents an efficacious platform for the conjugation of functional moieties. Meng et al. modified the H460 cell membrane through glycometabolism and linked it to the tripeptidyl peptidase 1 peptide, which can impede binding between PD-L1 and PD-1. Thereby, it inhibited tumor growth in vitro and in vivo [53].

Indeed, metabolic glycan labeling can also serve as a recognition target site in vivo. Nevertheless, the direct systemic administration of metabolic glycan precursors often lacks tissue- and cell-type selectivity, which can lead to dispersed labeling and low metabolic incorporation efficiency [54]. To enhance the selectivity and improve the efficiency of metabolic glycan labeling, advanced delivery systems, such as nanoparticles, are employed [55]. For example, Zhao et al. used 2-methacryloyloxyethyl phosphorylcholine (MPC), a hydrophilic monomer, and 2-lactobionamidoethy methacrylate (LAEMA), a polymer that can selectively identify liver cancer cells, to create pH-responsive Ac_4_ManNAz-releasing copolymer nanoparticles [56]. This strategy improved the labeling efficiency; it may have applications in high-resolution bio-imaging and targeted therapy.

Metabolic glycan labeling has limitations that circumscribe its clinical translation. The protracted temporal demands requisite for the incorporation of modified functional groups, coupled with the short modulation duration caused by glycan/membrane recycling, pose significant challenges [55]. Moreover, the integration of supraphysiological concentrations of unnatural precursors can perturb cellular homeostasis, including nonspecific interactions with cysteine residues. Further complicating the application of metabolic labeling is the dependency of metabolic activity on the cellular growth phase and the metabolic environment. Variability in these parameters begets inconsistent reproducibility of the metabolic modification, thus undermining the method’s reliability. Advancements in the refinement of metabolic glycan labeling are essential to ameliorate these concerns and enhance its viability for clinical diagnostic and therapeutic applications [57,58,59].

### 2.5. Genetic Engineering

Genetic engineering involves the editing of genes to facilitate the functional expression of cell membrane proteins, and it is a common technology for cell modification that operates via natural biosynthetic mechanisms [60,61,62,63].

Nucleic acids can be transported using chemical, biological, or physical methods [64]. Among them, lentiviral vectors are capable of transfecting both dividing and non-dividing cells, rendering them suitable for a range of gene delivery purposes [65]. This technology can successfully modify membranes while maintaining their activity and natural structure [66]. Compared to conventional noncomedies, the nanoparticles coated with genetically modified cell membranes could potentially improve targeted delivery to the nidus, boosting immune activity, and mitigating biotoxicity [18].

**Figure 6:**
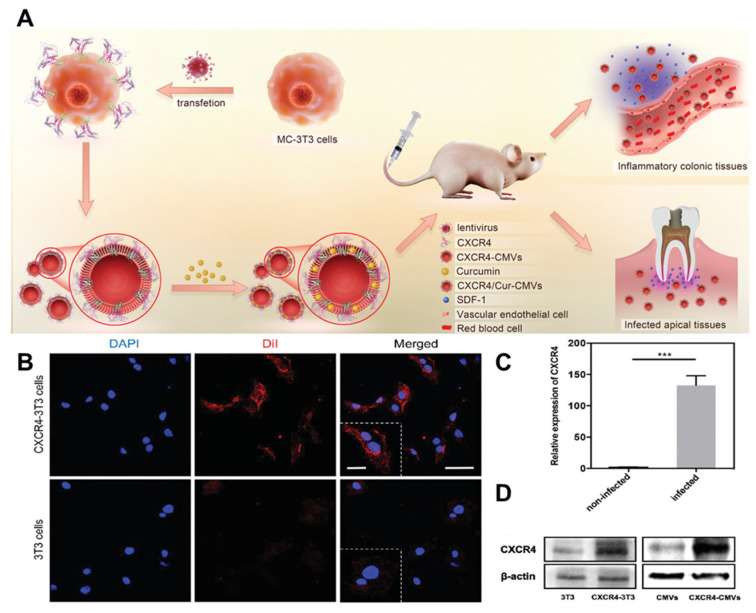
Development of genetic engineering. (**A**) The CXCR4-CMVs possess natural membrane surface characteristics. (**B**) After transfection with a lentivirus vector encoding the CXCR4/GFP chimeric protein, the fluorescence expression of membrane CXCR4 protein (red) in the transfection group’s 3T3 cells was significantly higher than that of the wild-type 3T3 cells. Scale bar = 50 µm. And a higher magnification. Scale bar = 20 µm. (**C**) The results of the RT-PCR analysis showed a significant increase in CXCR4 mRNA levels in the transfected group compared to the vehicle group (*** *p* < 0.001, n = 3). (**D**) The Western blot analysis revealed a significant increase in CXCR4 protein expression levels of CXCR4-3T3 and CXCR4-CMVs after lentivirus transfection. (RT-PCR: reverse transcription–polymerase chain reaction; CMVs: cell membrane vesicles; SDF-1: stromal cell-derived factor-1; DAPI: 4′,6-diamidino-2-phenylindole; Dil: 1,1′-Dioctadecyl-3,3,3′,3′-tetramethylindocarbocyanine perchlorate). Reprinted from [60] with permission from Wiley, open access, copyright 2021.

Coating nanoparticles with genetically engineered cell membranes has the potential of targeted delivery while reducing off-target effects. The C-X-C motif chemokine ligand 12 (CXCL12)/C-X-C chemokine receptor type 4 (CXCR4) binding plays a critical role in inflammatory chemotaxis [67]. Cell membrane vesicles (CMVs) enriched with CXCR4, generated through genetic engineering, can be employed as targeted delivery strategies to treat inflammatory disorders. For instance, Zhou et al. improved the targeting of central nervous system metastases by upregulating CXCR4 on neural stem cells using lentiviral transfection (Figure 6) [60]. This approach amplified targeting to the ischemic brain and enhanced delivery efficiency. Similarly, CXCR4 recombinant lentivirus was transfected into MC-3T3 cells, ultimately resulting in membrane CXCR4-rich MC-3T3 cells [68]. This provides a feasible innovative method for manufacturing targeted delivery systems for inflammatory sites. Moreover, genetic engineering can alter specific peptides on cell membranes to effectively cross the BBB and reach neoplastic cells. A liposomal extruder produced engineered artificial vesicles (EAVs) by directly squeezing HEK293T cells expressing the Angiopep-2 and TRP-PK1 fusion proteins [69]. These EAVs showed strong BBB penetration and superior targeting against GBM.

Equally, genetically engineered cell membranes can be used for therapeutic purposes. The binding of programmed cell death ligand 1 (PD-L1) and programmed cell death protein 1 (PD-1) leads to immune evasion in malignancy, establishing it as a crucial antitumor therapy [70]. To disrupt the immune tolerance pathway in the tumor microenvironment, indoleamine 2,3-dioxygenase-1 inhibitors were loaded on nanoparticles overexpressing PD-1. This led to noteworthy tumor killing and enhanced survival in mice carrying tumors [71]. Similarly, Chen et al. genetically engineered TC1P cell membrane-disguised nanoparticles [72]. The excessive PD-1 on the surface of the nanoparticle is bound to PD-L1 on the surface of lung cancer cells, thus producing anti-cancer effects.

Genetic engineering, while a potent tool for augmenting the characteristics of cell membrane-cloaked nanoparticles, is beset with inherent limitations. This methodology necessitates intricate and exacting protocols, with the feasibility of modulating a full spectrum of cell types being non-universal. Furthermore, genetic expression can exhibit variability across cellular generations, potentially leading to a diminution of target protein expression over time [73].

## 3. Nanomaterials for Engineered Cell Membrane Camouflage

An array of nanoscale constructs, encompassing both inorganic and organic nanoparticles, has garnered substantial attention across various biomedical arenas from therapeutic interventions to the screening of pharmacological agents attributable to their distinctive physicochemical properties [74]. Endeavors to augment both specificity and stability while concurrently conferring an intricate repertoire of functionalities have precipitated the genesis of novel nanostructures cloaked within engineered cell membranes. Functionally, these engineered cell membranes chiefly facilitate the precision-targeted conveyance of high-density therapeutic moieties. From the perspective of nanomaterial types, it reveals that magnetic nanoparticles account for a high proportion of the engineered cell membrane-camouflaged inorganic nanoparticles, whereas PLGA-based nanoparticles often epitomize the organic nanoparticles. Subsequent discourse will delineate them individually (Table 2).

### 3.1. Inorganic Nanoparticles

#### 3.1.1. Magnetic Nanoparticles

Magnetic nanoparticles typically refer to superparamagnetic Fe_3_O_4_ nanoparticles, which have garnered much research focus due to their potential application in magnetic resonance imaging (MRI), magnetic accumulation, and external magnetic field-guided targeting. Although they exhibit strong saturation magnetization for MRI, the low magnetization of each nanoparticle poses challenges in controlling motion effectively under moderate magnetic fields. Augmentation of particle size may enhance saturation magnetization, but it may also trigger a superparamagnetic-to-ferromagnetic transition, leading to nanoparticle aggregation in solution. To establish the magnetic moment of single clusters, which increase with increasing particle size while maintaining their superparamagnetic properties, Fe_3_O_4_ magnetic nanoclusters (MNCs) are constructed [145].

Anchoring tumor-targeting peptides onto the camouflaging membrane through glycometabolic engineering and orthogonal biological reactions may improve the targeting precision and drug delivery efficacy of nanomedicines. The RGD peptide is a highly effective and commonly utilized tumor-targeting peptide consisting of Arginine–Glycyl–Aspartate which binds specifically to integrin receptors situated on the tumor cell surface, promoting recognition for tumor targeting and subsequent transport into the cytoplasm. The cyclic variant, cRGD, precisely targets the over-expressed integrin αvβ3 receptor existing in the neovascular system. Consequently, it can cross the BBB. siRNA, also known as silent RNA, comprises a collection of small, double-stranded RNAs ranging from 21 to 25 nucleotides that possess the ability to silence certain genes in mammalian cells. Using siRNA to repress oncogenes or their transcription factors highlights an optimistic approach to managing cancer. Unfortunately, unmodified siRNA has a short half-life and a nonspecific biological distribution, which means that it will not be effectively delivered to the intended cell, tissue, or organ, thereby resulting in a lower-than-expected therapeutic effect. Zhang et al. capitalized on Fe_3_O_4_ MNCs’ targeting ability through external magnetic fields and employed the positive surface charge to bind siRNA, constructing a multi-functional siRNA delivery system [119]. To enhance the duration and the targeting ability, it was coated with a macrophage membrane with the RGD peptide. Camouflaging the nanomedicine with the macrophage membrane enables endogenous macrophages to recognize it as their own, thus extending its lifespan in the blood. The results have demonstrated that the nanoparticles effectively suppress target gene expression and inhibit tumor growth with minimal side effects. Similarly, Duan et al. utilized metabolic engineering and bioorthogonal reactions to modify cRGD on GBM cell membranes, followed by wrapping the nanoparticles to create a multimodal brain tumor imaging probe [105]. The nanoprobes’ core comprises the conjugated polymer with photoacoustic imaging properties and ultra-small iron oxide nanoparticles serving as contrast agents for MRI. The ability to target GBM is enhanced by the cRGD modification, allowing the nanoprobe to quickly and accurately locate and identify potential tumor margins.

**Figure 7:**
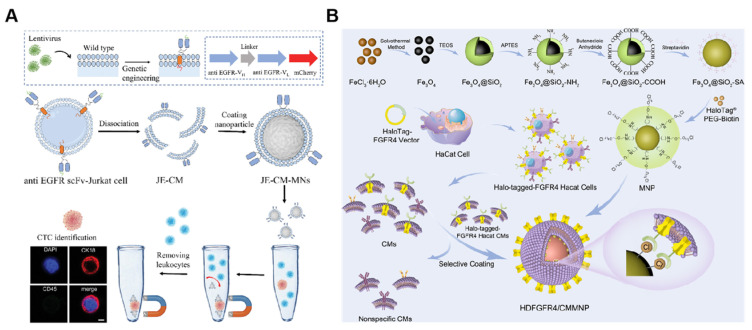
(**A**) Schematic illustration of overexpressing scFv of the EGFR antibody on the surface of the Jurkat cell membrane and cell membrane-camouflaged MNs. JE-CM-MNs were shown to maintain specific recognition of EGFR-positive CTCs. Scale bar = 5 µm. (ScFv: single-chain variable fragment; JE-CM: chimeric antibody membrane). Reprinted from [121] with permission from Wiley, copyright 2023. (**B**) The method of preparing HDFGFR4/CMMNPs. (FGFR4: fibroblast growth factor receptor 4). Reprinted from [127] with permission from ACS Applied Materials & Interfaces, copyright 2023.

Apart from their function of targeting tumor cells, magnetic nanoparticles disguised by engineered cell membranes can serve as a method to identify and isolate specific cell types. A crucial aspect involved in the formation of distant metastases is the shedding of circulating tumor cells (CTCs) from the primary tumor and their spread throughout the bloodstream. Accordingly, the identification of CTCs in peripheral blood is believed to be a highly potential biomarker for cancer metastasis, and the detection of CTCs holds significant importance in early cancer diagnosis and treatment response monitoring. However, the detection of CTCs in peripheral blood poses a well-known challenge because of their extremely low frequency with a proportion of less than one billion. One possible solution is to use magnetic nanoparticles with antibodies of CTCs anchored to their surface for selective enrichment from peripheral blood. Nevertheless, this tactic has inherent limitations as it cannot evade the nonspecific binding of white blood cells. To address this problem, Xie’s group used metabolic glycan-labeling white blood cell membranes to camouflage Fe_3_O_4_ MNCs and applied homology to exclude white blood cells, thereby decreasing the associated background [120]. In just 15 min, 90% of rare tumor cells could be isolated from whole blood without detecting any white blood cell background. This highlights that novel biomimetic magnetosomes have great potential for highly effective CTC enrichment. Chemical modification can impair the biological activity of antibodies; thus, Jiang et al. used genetic engineering to construct a chimeric antibody membrane on a single-chain variable fragment (ScFv) that overexpressed epidermal growth factor receptor (EGFR) antibodies on the human leukemia T lymphocyte (Figure 7A) [121]. The membrane was then applied to magnetic nanoparticles (JE-CM-MNs) to enrich CTCs. An ScFv is created by linking the variable regions of the heavy and light chains in an antibody with a 15–20 amino acid linker. Compared to classical antibodies, an ScFv has the advantages of small molecular weight, strong penetration ability, and weak antigenicity. Nanoparticles camouflaged with chimeric antibody membranes exhibited a binding affinity for extracellular EGFR more than 100 times higher, and the capture efficiency increased from 64.8% to 93.5% in artificial blood samples.

In addition to targeted recognition function, specific antibodies can be anchored on cell membranes through metabolic glycoengineering or genetic engineering, providing potential in immunotherapy. Additionally, Xie’s team developed a series of nanomedicines for tumor immunotherapy, in which specific antibodies were attached to cell membranes using metabolic glycoengineering or genetic engineering. Among these, Zhang et al. utilized an engineered white blood cell membrane containing azides to coat MNCs. They linked a major histocompatibility complex class I that carried the EG-7 tumor peptide and a co-stimulatory ligand anti-CD28 via a copper-free click reaction [120]. They constructed artificial antigen-presenting cells (aAPCs) to overcome the drawbacks of using natural APCs, such as their poor reproducibility and time-consuming production. The aAPCs possess the capability to not only augment CTLs but proficiently guide reperfusion CTLs into tumor tissue. The multifunctional aAPCs can be delivered by magnetic control and monitored by MRI based on the magnetization and T2 relaxation properties of MNCs. In another study, they generated a nanomedicine with a significant anti-cancer therapeutic effect by utilizing the possible synergism of ferroptosis and immunomodulation in malignancy. The MNCs served as the central component of the magnetosome, contributing to MRI guidance and targeting, regulating iron levels, and loading TGF-β. The surrounding cell membrane of the MNCs bound immune checkpoint antibodies via a click response. After being injected intravenously, magnetosomes accumulated and remained at the tumor site due to an external magnetic field and created an immunogenic tumor microenvironment. Simultaneously, the hydroxyl radical generated through the Fenton reaction involving iron ions induces tumor cell death [122]. In addition, magnetosomes can be designed as a high-performance cancer vaccine by retaining them in lymph nodes for effective immune activation. By adsorbing the Toll-like receptor (TLR) agonist CpG oligonucleotide onto the MNCs and attaching CD205 antibody to the engineered cell membrane mentioned above, the nanoparticles can be preferentially recognized by CD8^+^ DCs and subsequently activate CTLs [123]. This cancer vaccine’s anti-cancer efficacy and safety were demonstrated in five different tumor models, showing great promise for using this nanoparticle as a cancer vaccination. Additionally, macrophage modulation of cancer presents a promising therapeutic potential. Within the tumor microenvironment, CD47 on tumor cells associates with signal-regulatory protein α1 (SIRPα) on macrophages, transmitting a “don’t eat me” signal and polarizing into an M2 phenotype with the influence of colony-stimulating factors secreted by cancer cells. Genetically engineered overexpression of the SIRPα variant with a 50,000-fold increased affinity for CD47 on the macrophage membrane using genetic engineering can effectively block the CD47-SIRPα pathway. Therefore, magnetic nanoparticles with cell membrane coatings have been reasonably developed, which can help tumor-associated macrophages repolarize to the M1 phenotype, promote their phagocytosis, and activate T cells [124]. These nanoparticles significantly prolonged overall survival in mouse models of malignant melanoma and triple-negative breast cancer.

Magnetic nanoparticles coated with cell membranes are extensively used in the field of drug discovery, aside from direct therapy due to their distinctive targeting function at the biological interface. However, the binding site of drugs that target transmembrane proteins may reside in intramembrane domains. The random orientation of the cell membrane coating is inadequate for providing sufficient receptor density or effective binding to specific sites. Consequently, engineered cell membranes were employed to attain inner-to-outer-oriented cell membrane encapsulation of magnetic nanoparticles. For instance, Bu et al. prepared magnetic nanoparticles coated on the cell membrane with an inner-to-outer orientation by reacting the biotinylated outer surface of the cell membrane with Fe_3_O_4_ nanoparticles fixed with chain avidin to target and screen biologically active EGFR antagonists from natural products [125]. NHS-modified biotin was attached to membrane proteins through the EDC-activated acylation reaction. Similarly, Zhang et al. constructed magnetic nanoparticles by covalently coupling azide-functionalized cell membrane outer surfaces with alkyne-functionalized Fe_3_O_4_ nanoparticles to screen small molecule inhibitors [126]. HaloTag is a protein tag modified by halogenated alkane dehalogenase that can specifically covalently bind chlorinated alkanes. In their recent study, Bu et al. utilized genetic engineering to prepare a cell membrane expressing high-density fibroblast growth factor receptor 4 (FGFR4) with a HaloTag anchor [109]. They then coated it on magnetic nanoparticles (MNPs) through covalent site-specific mobilization between HaloTag and chloroalkane on magnetic nanoparticles to rapidly capture the potential FGFR4 antagonists (Figure 7B) [127]. High-density receptors improve drug screening sensitivity, while also broadening knowledge and techniques in membrane camouflaging technology.

#### 3.1.2. Semiconductor Nanoparticles

**Figure 8:**
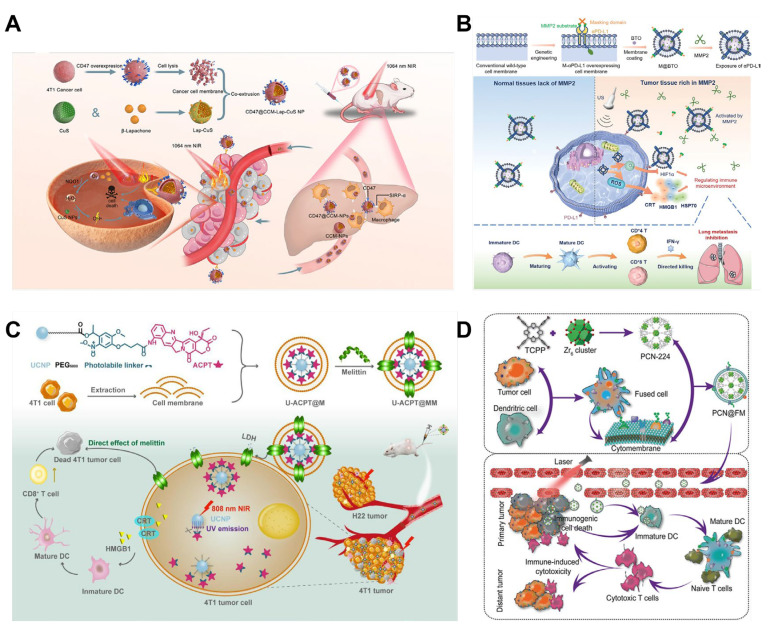
(**A**) The fabrication of CD47@CCM-Lap-CuS NPs. Under laser irradiation, a photothermal therapeutic effect is produced on the tumor tissue and Lap is released into breast cancer cells to generate H_2_O_2_. (Lap: *β*-Lapachone; NQO1: NAD (P) H:quinone oxidoreductase). Reprinted from [108] with permission from ACS Applied Materials & Interfaces, copyright 2023. (**B**) Schematic of structure of M-*α*PD-L1 overexpressing membrane-coated BTO nanoparticles. Following the delivery of M@BTO into the tumor microenvironment, the masking domain of the MMP2-sensitive peptide is cleaved and the binding domain of the antibody is exposed to exert the effect of blocking the PD-L1 receptor. Under US conditions, the BTO nanoparticles generate ROS to induce immunogenic cell death. (MMP2: matrix metallopeptidase 2; US: ultrasound; ROS: reactive oxygen species; PD-1: programmed death 1; PD-L1: programmed death ligand 1; HIF-1*α*: hypoxia-inducible factor 1-alpha; *α*PD-L1: anti-PD-L1 antibodies; HMGB1: high-mobility group box-1 protein; IFN-*γ*: interferon *γ*; HSP70: heatshockprotein70; CRT: calreticulin). Reprinted from [110] with permission from Wiley, copyright 2023. (**C**) Scheme of preparation of U-ACPT@MM. Upon irradiation at 808 nm, the short-wavelength UV emission of UCNPs breaks the photolabile linker, releasing ACPT, which is incorporated into the membranes of tumor cells. (ACPT: 9-aminocamptothecin; LDH: lactate dehydrogenase). Reprinted from [77] with permission from ELSEVIER, copyright 2023. (**D**) The process of synthesizing typical NPs involves a combination of immunotherapy and PDT. (FM: cytomembrane; DC: dendritic cell; TCPP: Tetrakis (4-carboxyphenyl) porphyrin; PCN: porous coordination network). Reprinted from [143] with permission from Wiley, copyright 2019.

Hollow mesoporous copper sulfide (CuS) nanoparticle is a proficient semiconductor material that possesses significant absorption and excellent photothermal conversion performance in the NIR-II region. Therefore, hollow mesoporous CuS nanoparticles show great potential as a multifunctional agent for cancer therapy. Additionally, CuS nanoparticles also demonstrate nanozyme activity and can catalyze the formation of cytotoxic hydroxyl radicals from H_2_O_2_. Zhan et al. utilized genetic engineering to construct cancer cell membranes that overexpressed CD47 (Figure 8A) [108]. Hollow CuS nanoparticles were coated with these membranes and demonstrated favorable PTT effects under NIR irradiation. Meanwhile, β-lapachone, a natural naphthoquinone compound loaded onto CuS nanoparticles, can produce a significant quantity of H_2_O_2_ within the tumor microenvironment. Afterward, CuS nanozymes catalyze H_2_O_2_ into hydroxyl radicals, resulting in chemodynamic therapy. Likewise, researchers coated hollow CuS nanoparticles loaded with doxorubicin onto a fusion membrane comprising RBCs and melanoma cells to achieve photothermal–chemical synergistic therapy for melanoma [109]. As opposed to naked CuS nanoparticles, the coated nanoparticles display targeted specificity toward the source cell line and a sustained blood circulation duration.

Sonodynamic therapy (SDT) is a procedure that utilizes ultrasound to activate a sonosensitizer to produce ROS, which effectively annihilates tumor cells. Ultrasmall barium titanate (BTO) nanoparticles are a piezoelectric catalytic material that can produce unbalanced charges when subjected to ultrasound treatment, resulting in a cascade oxidation–reduction reaction in water that generates ROS and O_2_. Tang et al. employed genetic engineering to affix PD-L1 antibodies onto the cell membrane via MMP2 substrates. They then wrapped the cell membrane around BTO nanoparticles to construct biomimetic vesicles (M@BTO NPs) (Figure 8B) [110]. The overexpressed MMP2 in the tumor microenvironment can cleave the substrates, releasing PD-L1 inhibitors. This nanoplatform can activate immunity through SDT, inhibit the PD-L1 pathway, and provide a reliable and secure method for improving the immune response. Likewise, Shi et al. developed a sonosensitizer named nP18 by conjugating purine 18 with DSPE-PEG_2000_-NH_2_ [111]. It was encapsulated within genetically engineered BHK-21 cell membranes. This nanoparticle serves for bimodal fluorescence/photoacoustic imaging-guided SDT. MMP2 cleaves and releases hyaluronidase on biomimetic membranes, facilitating the destruction of the tumor extracellular matrix to relieve hypoxia. This process consequently enhances the penetration of nanoparticles and enhances the effectiveness of SDT.

#### 3.1.3. Rare-Earth Upconversion Nanoparticles

Upconversion nanoparticles can convert NIR light into ultraviolet or visible light. Currently, light-induced release primarily depends on shortwave light [146]. However, NIR has become the preferred light source due to its higher tissue penetration depth and negligible side effects compared to ultraviolet light (UV) [147]. Therefore, upconversion nanoparticles hold tremendous potential as a therapeutic carrier that can be controlled by NIR. Based on this, lanthanide-doped upconversion nanoparticles (UCNPs) were bonded to the chemotherapeutic agent 9-aminotheophylline (ACPT) via UV-responsive linker and were covered with the 4T1 tumor cell membrane with melittin (U-ACPT@MM) (Figure 8C) [77]. Upon 808 nm irradiation, the UV radiation emitted by UCNPs cuts the photo-induced linker and releases ACPT. The cell membrane has the ability to target 4T1 tumors homogeneously as well as boost the concentration of chemotherapy drugs within the tumor. This ultimately leads to a considerable inhibition of both 4T1 tumors and pulmonary metastases with reduced adverse effects in vivo.

In a comparative analysis with traditional down-conversion fluorescent probes, including organic dyes and quantum dots, UCNPs exhibited remarkable chemical and photonic merits. These include their sharply defined emission maxima, low cytotoxicity, and robust photostability, which together render UCNPs highly suitable for bioimaging applications. Folate receptors have been widely used for cancer targeting, known to be overexpressed across a plethora of cancer types. Lang et al. have engineered RBC membranes functionalized with DSPE-PEG-modified folic acid to encapsulate hexagonal β-NaYF_4_:Er^3+^, Yb^3+^ UCNPs, yielding folate receptor-targeted UCNPs (FA-RBC-UCNPs) [75]. FA-RBC-UCNPs have shown augmented tumor localization, advancing their potential as precision agents for tumor upconversion luminescence imaging.

#### 3.1.4. Metal–Organic Framework Nanoparticles

Metal–organic frameworks (MOFs) are crystalline materials composed of metal clusters or ions with organic ligands. They possess a highly ordered pore structure and a large specific surface area, making them applicable in the field of drug delivery [148]. Photodynamic therapy (PDT) employs light of specific wavelengths for exciting photosensitizers to generate ROS to eliminate cancer cells. Embedding photosensitizers into MOFs can eliminate the drawback of self-quenching of excited states of photosensitizers and enhance the diffusion of generated ROS [149]. Liu et al. synthesized porphyrin-based Zr-MOF (PCN-224) by using zirconyl chloride octahydrate and photosensitizer tetrakis (4-carboxyphenyl) porphyrin as raw materials (Figure 8D) [143]. The study found that encapsulation of PCN-224 MOF using the fusion membrane from DCs and cancer cells is capable of simulating two immune activation pathways, thus providing an effective and long-lasting immunotherapy for primary tumors. In a separate project, this research group implemented this nanomaterial to develop imageable cancer vaccines. Compared to other cellular vaccines, using this vaccine that excludes genetic nanomaterial offers superior biosafety, simpler mass production, and prolonged storage time [144].

#### 3.1.5. Noble Metal-Based Nanoparticles

Au nanoparticles, with their intrinsic hollow architecture, exceptional photothermal conversion proficiency, and commendable biocompatibility, have seen prolific utilization in biomedical applications [150]. A pivotal innovation in enhancing the therapeutic index of these particles is their encapsulation within engineered cell membranes. This strategy ingeniously addresses and mitigates the potential attenuation of antitumor activity attributable to the native immune evasion deficits associated with bare Au nanoparticles. Zhu et al. have exemplified this approach by adorning red blood cell membranes with target-specific epithelial cell adhesion molecule (EpCam) antibodies, directing their homing capabilities to 4T1 cancer cells [112]. Encased within these engineered cell membranes are Au nanocages loaded with the paclitaxel. Upon being subjected to an 808 nm laser irradiation, these membrane-cloaked Au nanoparticles rapidly transduce light into heat and the paclitaxel is released. This dual-action modality culminates in a substantial induction of cancer cell mortality.

Membrane hybridization can enhance the functionality of Au nanoparticles. For instance, Hao et al. capitalized on the innate homing propensities of both platelets and neutrophils toward CTCs and tumor-derived exosomes [113]. A carefully crafted hybrid cell membrane was employed to cloak doxorubicin and indocyanine green (ICG) co-loaded within the confines of Au nanocages. Notably, these bionic nanocarriers (PNMAuDIs) adeptly intercepted CTCs, thereby mitigating their potential to seed distant metastases. They also proficiently neutralized the promigratory cues of tumor-derived exosomes, which play a surreptitious role in priming pre-metastatic niches. Such findings not only underline the adept engineering of these nanosystems but also project a novel framework for attenuating metastatic breast cancer. Similarly, an approach lies in the manufacturing of an acoustic Au nanowire, sheathed within a hybrid membrane that synergizes the biological attributes of RBCs and platelets [114]. This artful design confers upon the resultant nanowire a profound capability to mimic the complex interactive behavior of natural cells, transmuting it into a formidable biomimetic nanorobot. The nanorobot thus configured can embark on a multifaceted mission of biological decontamination, effectuating the eradication of pathogenic bacteria and neutralization of toxins.

#### 3.1.6. Nonmetal-Based Nanoparticles

The layered semiconductor material, black phosphorus (BP), is an emerging focus in two-dimensional material research due to its distinctive structure and intriguing optical properties. Its folded structure and negatively charged surface enable the electrostatic adsorption of a substantial number of positively charged drugs [151]. Black phosphorus quantum dots (BPQDs) display remarkable optical and electronic characteristics beyond the present two-dimensional features of BP owing to quantum constraints, edge effects, and large surface area [152]. A BPQD dispersion was exposed to 808 nm light, resulting in a temperature increase from 18.3 °C to 50.1 °C in just 7 min [153]. The corresponding photothermal conversion efficiency was determined to be 28.4% [154]. Nonetheless, it should be noted that BPQDs are susceptible to oxidative degradation when in contact with oxygen and water. To overcome this issue, BPQDs underwent a coating process involving a hybrid platelet and osteosarcoma membrane to improve their stability. This technique has led to a noteworthy advancement in the PTT for osteosarcoma [103].

Mesoporous silica nanoparticles (MSNs) are highly desirable as nanocarriers because of their large specific surface area, excellent thermal and chemical stability, great biocompatibility, and ease of surface modification [155,156]. The modification of biodegradable MSNs using engineered cell membranes via lipid insertion has been proven to be an effective and safe method for delivering siRNA to liver cells. This nanoparticle can suppress the expression of the target PCSK9 gene to treat non-alcoholic fatty liver disease [24].

### 3.2. Polymeric Nanoparticles

#### 3.2.1. Drug-Carrying Polymeric Nanoparticles

Traditional drug delivery methods require multiple daily injections to achieve the desired therapeutic effects. To improve patient compliance and convenience, sustained-release formulations have been developed that provide controlled release over an extended period of days or months. Among them, polymers like PLGA have been widely employed as controlled-release drug carriers thanks to their biodegradability and high biocompatibility [157]. The release kinetics of PLGA nanoparticles can be modulated by varying the lactic acid-to-glycolic acid molar ratio and polymer molecular mass.

**Figure 9:**
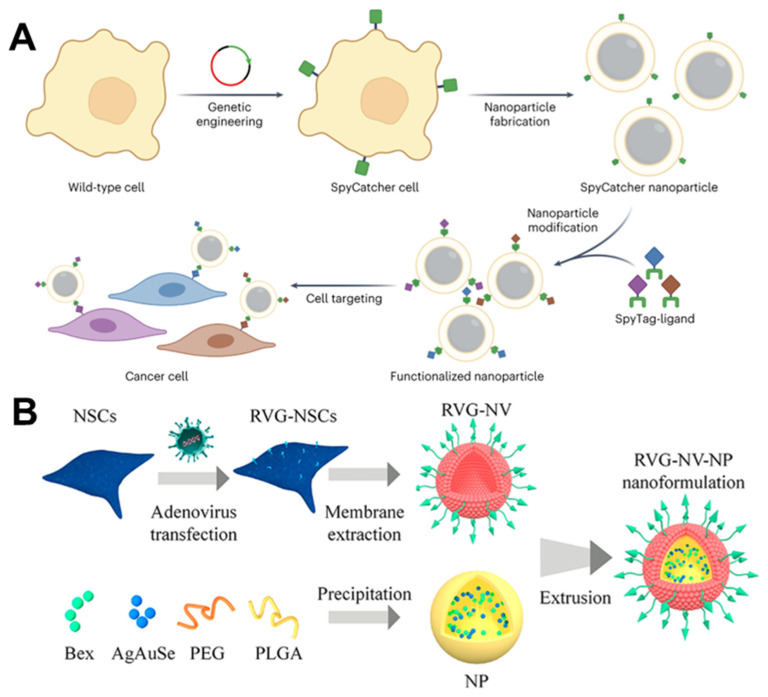
(**A**) The membrane from the SpyCatcher-expressing cells can be coated onto nanoparticle cores to create CNPs. These CNPs can then be functionalized with SpyTag-labeled ligands to enhance their functionality in a modular way. Reprinted from [89] with permission from Springer Nature, copyright 2023. (**B**) Schematic illustration of using adenovirus containing GFP and Lamp2b RVG to induce NSCs to express specific Lamp2b. (Lamp2b: glycoprotein 2b; NSC: neural stem cell; Bex: bexarotene; RVG: rabies viral glycoprotein). Reprinted from [102] with permission from American Chemical Society, copyright 2023.

Soluble proteins are typically modified on cell membranes by genetically fusing them with another transmembrane protein, which is more challenging. These fusion proteins must be optimized for specific circumstances to prevent misfolding, reduced function, and low expression levels due to steric hindrance. Krishnan et al. developed a strategy to decouple the expression of the transmembrane anchor and the soluble ligand using the SpyTag–SpyCatcher binding pair, providing a modular approach for cell membrane functionalization (Figure 9A) [89]. The modularity of this method was confirmed by using three different SpyTag-labeled targeted molecules.

Drug delivery to the central nervous system is more rigorous than to other areas [158]. Hence, optimizing systemic blood circulation, BBB penetration, and target cell localization is critical for treating central nervous system disorders. The engineered membrane-coating strategy can extend the half-life of nanomaterials in the bloodstream and improve the BBB-crossing and -targeting capabilities. Nanoparticles coated with RBC membranes have notable advantages, including heightened biocompatibility, superior ability to evade the immune system, circulation time beyond 24 h, and a certain aptitude for penetrating tumors. According to this, Lv et al. encapsulated a dextran polymer core loaded with the neuroprotective agent NR2B9C and modified with ROS-responsive groups using the RBC membrane and then inserted a stroke-homing peptide connected with DSPE allowed for specific targeting ability. This approach addresses the issues of short lifespan, lack of precision targeting, and inefficient drug release of delivery carriers in traditional ischemic stroke medicine [100]. The candoxin (^D^CDX) peptide (FKESWREARGTRIERG) derived from candoxin shows a high binding affinity with highly expressed nicotinic acetylcholine receptors on the BBB. Therefore, brain-targeted delivery of chemotherapy drugs can be achieved by modifying the RBC membranes with the ^D^CDX peptide to encapsulate PLGA nanoparticles loaded with doxorubicin. Although inserting a lipophilic substance into the membrane is convenient, the ^D^CDX peptide is a targeted ligand with a strong positive charge, which tends to adhere to negatively charged cell membranes. Thus, direct insertion will result in damage to its targeted delivery ability. Cai et al. first inserted DSPE-modified streptavidin into the RBC membrane and then bound the biotinylated ^D^CDX to the surface to avoid the ionic interaction between the ^D^CDX peptide and the RBC membrane [101]. Both in vitro and in vivo studies have confirmed the ability of this nanomedicine to cross the BBB and exhibit remarkable brain-targeting effects. Rabies virus glycoprotein (RVG) is a glycopeptide that specifically targets cerebral endothelial and nerve cells. The construction of neural stem cells that express RVG to a high degree through genetic engineering and the utilization of these cells to entrap PEG/PLGA nanoparticles loaded with the Alzheimer’s disease medicine bexarotene and NIR-Ⅱ fluorescent AgAuSe quantum dots can serve as an observation of brain drug delivery nanoprobe (RVG-NV-NP) for Alzheimer’s disease in situ (Figure 9B) [102].

Engineering cell membranes can be designed to target inflammatory sites for treating inflammation-related diseases. For instance, Deng et al. employed genetic engineering to construct a RAW264.7 macrophage that expresses TLR4. TLR4 is a crucial pathway for macrophages to identify pathogenic bacterial endotoxin receptors and activate innate immunity. The engineered cell membrane encloses antibiotic-loaded natural polymeric silk fibroin nanoparticles, successfully addressing the issue of ineffective antibacterial and immune regulation synergy present in conventional periodontitis drug therapy [137]. Furthermore, the inflamed regions’ high secretion of pro-inflammatory cytokines has the potential to modify circumscribed surface markers of vascular epithelial cells. Targeting anti-inflammatory agents to the affected area through characteristic molecules presents a promising strategy. Vascular cell adhesion molecule-1 (VCAM-1) is upregulated in inflamed endothelial cells and is a common characteristic. Thus, genetic engineering was employed to modify mouse leukemia C1498 cells, obtaining cell membranes with abundant VCAM-1 ligand expression. PLGA nanoparticles loaded with dexamethasone can specifically target inflammatory endothelial cells and effectively alleviate symptoms in a mouse model of endotoxin-induced pneumonia [88].

Photothermal therapy (PTT) is a treatment that involves the use of photothermal agents (PTAs) to convert NIR light into heat, which destroys cancerous cells [159]. It is generally believed that when tumor tissue is exposed to a temperature above 41 °C, irreversible tumor cell death can occur due to protein destruction. Simultaneously, the thermal pressure wave propagates as ultrasound within the tissue and can be monitored by external ultrasound transducers to generate tissue images, namely photoacoustic imaging (PAI) [160]. ICG is a small fluorescent molecule with excellent photothermal conversion capability that has been approved by the U.S. Food and Drug Administration [161]. PLGA is commonly utilized as a carrier to load ICG, which produces nanoparticles exhibiting a strong photothermal response and outstanding fluorescence/photoacoustic imaging abilities. To further improve the efficacy of photothermal therapy, active targeted modification technology can be employed for an improved PTT effect. Chen et al. utilized cancer cell membranes to encase PLGA nanoparticles containing ICG for homologous targeting [90]. To decrease non-specific binding between nanomedicines and serum proteins as well as extend circulation duration, DSPE-linked PEG was inserted into the MCF-7 cell membrane via lipid insertion, effectively enhancing the functional cell membrane’s targeting efficacy. Moreover, the T-cell membrane comprises immune recognition proteins, particularly T-cell receptors (TCRs), that permit activated T cells to accurately recognize and attach to tumor cells. However, depending solely on TCRs poses a potential danger of off-target effects. Consequently, the bicyclo [6.1.0] nonyne motif was modified on the tumor membrane via metabolic glycogineering as an artificial receptor-like target [91]. This targets an in situ bioorthogonal reaction with azide groups on T cells. The resulting dual-targeting approach enhances nanoparticle accumulation in malignant lesions and significantly improves PTT efficacy in vivo.

Engineered cell membrane-camouflaged nanoparticles serve not only as a targeting or recognition strategy but also as an avenue to directly connect therapeutic molecules to achieve desired therapeutic functions, such as blocking antibodies. The severe acute respiratory syndrome coronavirus 2 (SARS-CoV-2) presented an unparalleled threat to global public health [162]. During infection, the spike-like proteins (S proteins) interact with various membrane components including heparin and heparan sulfate (HS), ultimately facilitating entry into host cells. Based on this research, Ai et al. added azido groups (-N_3_) to host cell membranes through glycol expression, and then coated them onto PLGA nanoparticles [92]. These were then coupled with dibenzocyclooctyne group-functionalized heparin to synthesize “cellular nanosponges” with a higher density of heparin. The study indicated that this nanosponge has an enhanced capacity to bind to spike glycoprotein, thereby blocking virus entry into cells and exhibiting an inhibitory effect on virus infections. Significant progress has been made in treating certain solid tumors by inhibiting the activity of immune checkpoints. However, GBM has limited clinical success due to the obstruction posed by the BBB and the highly immunosuppressive tumor microenvironment. Nanomaterial-assisted therapy for GBM presents an opportunity to address relevant concerns. For instance, enhancing PD-1 expression on macrophages through genetic engineering and encapsulating rapamycin-loaded PLGA nanoparticles can improve immune checkpoint blockade therapy [93]. Unlike unmodified nanoparticles, these engineered cell membrane-camouflaged nanoparticles can cross the BBB and have successfully exhibited anti-GBM effects in vivo.

#### 3.2.2. Stimulus-Responsive Polymeric Nanoparticles

Stimulus-responsive polymers represent an innovative class of drug delivery vectors, which are characteristically endowed with the capacity to undergo a cascade of reactions in response to the unique biological microenvironment or external stimuli. Manifesting through a range of dynamic responses, such as chemical bond cleavage or morphological change, these polymers are increasingly harnessed within the biomedical domain, holding promise for advancing targeted therapeutic interventions [163].

Small-molecule PTAs display inadequate photothermal stability, whereas NIR stimulus-responsive polymers exhibit an easily modifiable chemical structure, adjustable NIR absorption, and superb biocompatibility. Su et al. integrated 1,2-Dipalmitoyl-sn-glycero-3-phosphocholine (DPPC) with a melting transition temperature of 41.5 °C into the shell enveloping the poly (caprolactone)-ester endcap polymer (PCL) [138]. They were encapsulated by inserting the lipophilic cyanine dye 1,1-dioctadecyl-3,3,3,3-tetramethylindotricarbocyanine iodide (DiR) into the membrane of RBCs. Under NIR exposure, the resultant temperature increase can damage the membrane and disturb the configuration of nanoparticles, allowing the discharge of loaded chemotherapy drugs. The nanoparticles effectively suppressed the propagation of primary tumors and curbed over 98% of lung metastasis in vivo. Polydopamine (PDA) is another substance used for PTT, which can release the loaded drug quickly when exposed to NIR. In their study, Guo et al. employed PDA to load glucose oxidase and a hypoxia-activated prodrug [139]. The latter was encapsulated in human osteosarcoma cell membranes that were modified with cRGD peptides. Following internalization by tumor cells, the nanoparticles significantly suppress tumor growth in a hypoxic environment by integrating starvation therapy, hypoxia-activated chemotherapy, and PTT.

pH-sensitive polymer nanoparticles are widely used in specific microenvironments due to their unique release characteristics. For instance, excessive activation of osteoclasts, causing osteoporosis, is attributed to the increase in the receptor activator of nuclear factor-κB ligand (RANKL). To neutralize RANKL and release synthetic parathyroid hormone (PTH 1-34) within the bone microenvironment, a pH-sensitive chitosan nanogel was covered with the membranes of genetically engineered bone mesenchymal stem cells overexpressing RANK and CXCR4 [140]. Owing to its delicate release properties, lengthier circulation time, and drug accumulation in bones, medicinal dosage and frequency can be markedly reduced with satisfactory therapeutic outcomes. Similarly, Yin et al. employed a cationic nanocore (BSPC) constructed from membrane-penetrating helical polypeptide (P-Ben) and Sav1 siRNA (siSav1), utilizing charge-reversal intermediate layers comprising poly (L-lysine)-cis-aconitic acid (PC), combined with platelet–macrophage hybrid membranes (HMs) to facilitate the targeted delivery to cardiomyocytes [36]. In the presence of an inflammatory microenvironment characterized by high acidity, the initiation of PC charge reversal was observed, resulting in the gradual degradation of both the HM and PC layers and subsequently exposing the P-Ben/siSav1 nano complexes. Subsequently, by inhibiting the Hippo pathway, cardiomyocyte regeneration was induced. This remarkable study introduces a biomimetic strategy that effectively tackles multiple systemic barriers encountered during siRNA delivery to the myocardium, thus positioning it as a promising gene therapy approach for addressing heart injuries.

Mitochondria account for approximately 90% of intracellular ROS production, making ROS-responsive polymers an attractive candidate for targeted delivery and release of therapeutic substances in mitochondria [164]. To this end, Zou et al. proposed a unique hybrid membrane comprised of cancer cells and mitochondria that effectively camouflages the nanoparticle ROS-responsive poly (ethylene glycol)-poly (4-(4, 4, 5, 5-Tetramethyltetramethyl-1, 3, 2-dioxaborolan-2-yl) benzyl acrylate) loaded with Gboxin [33]. This innovative nano platform allows for the non-invasive, targeted delivery and release of Gboxin to the mitochondria of GBM cells. This inactivates ATP synthesis within the mitochondria, ultimately leading to mitochondrial-mediated tumor cell apoptosis.

### 3.3. Lipid-Based Nanoparticles

**Figure 10:**
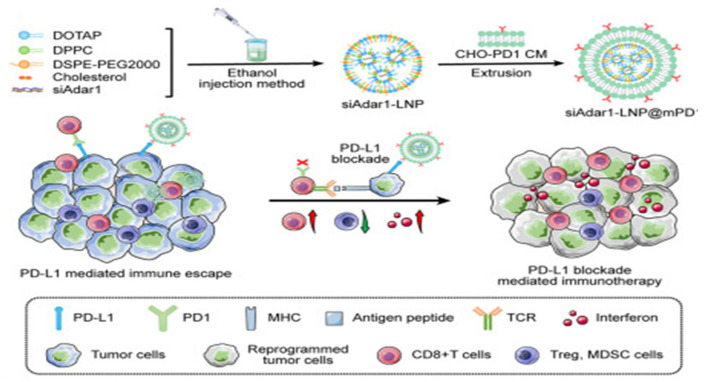
The LNP was loaded with synthetic ADAR1-siRNA with high encapsulation efficiency. Afterward, a layer of GECM with PD1 overexpression was coated onto the siAdar1-loaded LN. (siAdar1-LNP@mPD1: a layer of GECM with PD1 overexpression was coated onto the siAdar1-loaded LNP; siAdar1: small interfering RNA against ADAR1; LNP: lipid nanoparticle; ADAR1: adenosine deaminases acting on RNA; ICB: immune checkpoint blockade; GECMs: genetically engineered cell membranes; siAdar1-LNP@mPD1: siAdar1-loaded LNP was coated with a layer of the GECM with PD1 overexpression; siAdar1-LNP: LNPs to deliver ADAR1-targeted siRNA; MHC: major histocompatibility complex). Reprinted from [99] with permission from Cell Press, copyright 2023.

Nanosuspensions (NSs) represent a novel advancement within the realm of nanomedicine delivery systems, emerging as a new dosage form specifically designed for insoluble drugs. Characterized by their straightforward synthesis and high reproducibility, NSs markedly enhance the therapeutic potential of compounds hindered by insolubility. Fan et al. used paclitaxel and sodium deoxycholate as raw materials and employed the ultrasonic precipitation method to synthesize a paclitaxel nanosuspension ((PTX)NS) [98]. The targeted peptide ^D^WSW (DSDYDPDGDWDSDW) was encapsulated in the C6 cancer cell membrane (CCMs) through lipid insertion to obtain ^D^WSW-CCM-(PTX)NS. The ^D^WSW-CCM-(PTX) NS can effectively penetrate the BBB and increase drug accumulation at the tumor site after administration. Similarly, lipid nanoparticles carrying siRNA also have great application prospects. Zhang et al. encapsulated siRNA targeting ADAR1 that may impede the effectiveness of ICB therapy within a lipid nanoparticle-based siRNA delivery system, which was encapsulated within PD-1-overexpressing cell membranes (Figure 10) [99]. These nanoparticles (siAdar1-LNP@mPD1) efficiently silenced the ADAR1 gene and coupled with PD-L1 on cancer cells, providing them with a potent antitumor immune response.

Wei et al. synthesized a photothermal material (PBTPBFTDPP) and subsequently self-assembled it with the amphiphilic polymer DSPE-PEG_2000_ to produce water-soluble nanoparticles [141]. These nanoparticles demonstrated outstanding photothermal conversion ability, with a photothermal conversion coefficient of 53.6%, and exceptional photothermal stability. The researchers selected upregulated transferrin receptors as targets in metastatic and drug-resistant malignancies. Hence, they obtained cell membranes displaying high levels of transferrin from human embryonic kidneys via genetic engineering and coated them onto these nanoparticles. The modified nanoparticles exhibited superior targeting ability and a potent PTT effect on human liver cancer.

## 4. Summary and Outlook

To imbue cell membranes with sophisticated functionalities, techniques in cell membrane engineering have been devised and extensively utilized [165]. These methods represent a pioneering fusion of synthetic nanomaterials with biological systems and enrich nanoparticles with a spectrum of biological capabilities. Within this review, we provide a concise collation of the predominant techniques employed in cell membrane engineering including lipid insertion, membrane hybridization, direct chemical modification, metabolic glycan labeling, and genetic engineering. Each methodology is dissected to unravel its underlying principles, alongside a critical examination of its merits and limitations, with a focus on the novel functionalities elicited in nanoparticles through cell membrane engineering. Grounded on this framework, we delineate the advancements in research on nanoparticles camouflaged with engineered cell membranes across various biomedical domains. This review not only augments comprehension of the field’s progression but also illuminates the path toward more profound investigations.

Despite the dynamism of research efforts, the field of engineered cell membranes remains nascent, confronting limitations related to the depth of understanding about cell membranes and manipulation tools. First, the intrinsic dynamics and turnover of cell membranes pose challenges; the propensity for the loss of externally added modification molecules is one such issue during the engineering and preservation processes, contributing to the unstable nature of cell membrane camouflage methodologies. Additionally, exogenous substances introduced during membrane engineering may exert uncharacterized impacts upon normal cellular metabolism, invoking apprehensions about the long-term biosafety and biocompatibility requisite for subsequent in vivo applications. Meanwhile, it is necessary to expunge potentially deleterious agents and unwarranted proteins that arise through the engineering process, which is a problem that demands prompt resolution. Furthermore, the technological maturity for producing nanoparticles encapsulated by engineered cell membranes is wanting. The number of derived cell membranes is contingent on the nature of progenitor cells and the sophistication of purification techniques, leading to poor replicability. Prevailing methodologies and protocols largely cater to research within laboratory settings, significantly circumscribing the potential for industrial-scale deployment of this technology and impeding its translation into clinical practice. Lastly, the establishment of regulated industry benchmarks has yet to occur. As with the broader category of nanomedicines, comprehensive methods for evaluating the performance of cell membrane-cloaked nanomaterials, including their pharmacodynamics and pharmacokinetics, remain to be instituted.

Looking forward, the initial imperative is to deepen our understanding of the interplay between synthetic molecules and cellular functions, tailoring engineering tactics for specific cell types and their intended applications. For instance, insights into how the cholesterol content in cell membranes influences the activity of encapsulated nanozymes warrant methods that enhance transmembrane molecular transport [142]. Moreover, since modified molecules on cell membranes can be subject to endocytosis or secretion, devising monitoring strategies to track the efficiency and dynamics of modifications is equally vital. The second challenge lies in the advancement of highly biocompatible methods capable of selectively integrating exogenous molecules onto cell surfaces. This effort necessitates progress in multidisciplinary foundational research within biology and chemistry to expedite the extension of our cell membrane engineering toolkit. The novel metabolic labeling techniques, which permit the attachment of diverse functional groups to membranes, are exemplary of this direction. Furthermore, audacious implementation of state-of-the-art technologies and pioneering discoveries should be encouraged within this realm. Thirdly, concurrent with these research endeavors, the commercial scalability of this technology also must be addressed to improve every facet of the production and preservation processes. For instance, the integrity of membranes can be jeopardized by conventional cryostorage; thus, the development of novel cryoprotectants will mitigate such damage without introducing biotoxicity. Fourthly, it is essential to diversify the array of biomimetic membranes and nanomaterials and broaden their application horizons. For example, advancing the development of extraction and encapsulation techniques for membranes sourced from exosomes [166], bacteria [167], and viral capsids [168] is important. Such technologies could pave the way for engineered cell membrane-camouflaged hydrogels, nanofibers, and other nanomaterials. The potential of these engineered membranes extends far beyond their current applications, envisioning their deployment in diverse sectors, including osmotic energy generation and the burgeoning industry of smart textiles. Lastly, leveraging existing databases, there is an opportunity to construct dedicated databases for engineered biomimetic membranes. These databases would empower researchers in the meticulous design of optimal modification strategies. When coupled with artificial intelligence, a paradigm shift toward custom-tailored biomimetic membranes can be foreseeable, and it will accelerate the innovation cycle within this field. Bridging disciplines, these engineered cell membrane-camouflaged nanomaterials could stand at the vanguard of sustainable and advanced technology, harnessing biological principles to enhance their functionality and integration within various environmental and technological contexts.

## Figures and Tables

**Table 1:** Summary of cell surface engineering strategies.

Engineering Strategies	Principle	Advantages	Limitations
Lipid insertion	Hydrophobic interactions and fluidity of lipid membranes	Easy to operate; Low time cost	Weak binding force and instability; No specificity
Membrane hybridization	Fluidity of lipid membranes	Composite multiple membrane functions	Difficulty in determining fusion strategies; Introducing unnecessary molecules; Low accuracy and repeatability
Direct chemical modification	Forming covalent bond connections, such as acylation reactions	Stable; Persistent	Damage to membrane protein function; No specificity
Metabolic glycan labeling	Non-natural glycan metabolism	High labeling efficiency; Strong covalent binding in biological orthogonal reactions; Mild conditions; Specificity	Long operation time; Existence of non-specific reactions
Genetic engineering	Gene editing and gene transduction	The highest specificity; Easy to mass produce; Accurate regulation; Maintaining protein biological activity	Complex process, labor-intensive, and high technical requirements; Limited modifiable cell types; Unstable expression level

**Table 2:** Engineered cell membrane-camouflaged nanoparticles and their applications.

Nanomaterials	Name	Membrane Source	Engineering Methods	Applications	Ref.
Upconversion nanoparticles (UCNPs)	FA-RBC-UCNPs	Red blood cell (RBC)	Lipid insertion	Tumor imaging	[75]
	RBC-UCNPs	RBC	Lipid insertion	Tumor imaging	[76]
	U-ACPT@MM	4T1 tumor cell	Lipid insertion	Cancer therapy	[77]
Bismuth nanoparticles	F-RBC bismuth nanoparticles	RBC	Lipid insertion	Cancer therapy	[78]
a-dextran loading Dox/Lex	Ang-RBCm@NM-(Dox/Lex)	RBC	Lipid insertion	Cancer therapy	[79]
Dextran-grafted-poly (histidine) copolymer	DH@ECm	Erythrocyte cancer cell	Membrane hybridization	Cancer therapy	[80]
Dextran (m-dextran) polymer nanoparticles	tP-NP-rtPA/ZL006e	Platelet (PLT)	Direct chemical modification	Ischemia therapy	[81]
PLGA	HA-mRNA-NP	B16-HA cell	Genetic engineering	Gene therapy	[82]
	Man-RBC-NPhgp	RBC	Lipid insertion	Cancer vaccines	[83]
	iE–RBCmGA/PLGA	RBC	Lipid insertion	Cancer therapy	[84]
	TRAIL-Dox-PM-NV	PLT	Lipid insertion	Cancer therapy	[85]
	TM-CQ/NP	LX2 cell	Genetic engineering	Stroke therapy	[86]
	CMNPs	Neural stem cells	Genetic engineering	Cancer therapy	[68]
	MSC-PD-L1 NPs	Mesenchymal stem cells	Genetic engineering	Inflammation therapy	[87]
	VLA-DEX-NP	VCAM-1	Genetic engineering	Cancer therapy	[88]
	RGD-RBC-NC (DTX)	RBC	Lipid insertion	Lipid insertion	[25]
	MΦ-NP (L&K)	Macrophage	Cancer therapy	Genetic engineering	[29]
	mKate2-NPs	HEK293-SC cell	Genetic engineering	Cancer therapy	[89]
	ICNPs	MCF-7 cell	Metabolic glycan labeling	cancer therapy	[90]
	n_3_-tINPs	t cell	Metabolic glycan labeling	Cancer therapy	[91]
	HP-NS	Host cell	Genetic engineering	Virus therapy	[92]
	PD-1-MM@PLGA/RAPA	Macrophage	Genetic engineering	Cancer therapy	[93]
	NP-R@M-M	Cancer cell	Lipid insertion	Cancer vaccines	[94]
Solid lipid nanoparticle	T7/NGR-RBCSLNs	RBC	Lipid insertion	Cancer therapy	[95]
	Lipo-Ce6/TPZ@MH	PLT and RBC	Membrane hybridization	Cancer therapy	[96]
	Lp-KR-CCM-A	4T1-Fluc cancer cells	Genetic engineering	Cancer therapy	[97]
	CCM-(PTX) NS	C6 cancer cell	Genetic engineering	Cancer therapy	[98]
	RBC-NPs	RBC	Lipid insertion	Cancer therapy	[30]
	siAdar1-LNP@mPD1	Tumor cell	Genetic engineering	Gene therapy	[99]
Dextran polymer core loaded with neuroprotective agent NR2B9C	SHp-RBC-NP	RBC	Lipid insertion	Ischemic stroke therapy	[100]
Streptavidin–PEG_3400_–DSPE and biotin–PEG_3500_–DCDX	D CDX-RBCNPs	RBC	Lipid insertion	Cancer therapy	[101]
AgAuSe quantum dots (QDs)	RVG-NV-NPs	Neural stem cell	Genetic engineering	Alzheimer’s Disease therapy	[102]
Black phosphorus quantum dots (BPQDs)	BPQDs-DOX@OPM	PLT and osteosarcoma	Membrane hybridization	Cancer therapy	[103]
Ultra-small Fe^0^ nanoparticle (Fe^0^NP)	GOx-Fe^0^@EM-A	RBC	Lipid insertion	Cancer therapy	[104]
	cRGD-CM-CPIO	Tumor cell	Metabolic glycan labeling	Tumor imaging	[105]
Magnetic beads	HM-IMBs	White blood cell (WBC) and PLT	Membrane hybridization	Circulating tumor cell detection	[106]
Dispersed starch and PEG diacid-coated magnetic nanoparticles	FVDSPM	RBC	Lipid insertion	Cancer therapy	[107]
Hollow CuS nanoparticles	CD47@CCM-Lap-CuS NP	Cancer cell	Genetic engineering	Cancer therapy	[108]
	DCuS@ [RBC-B16] NPs	RBC and melanoma cell	Membrane hybridization	Cancer therapy	[109]
BTO nanoparticle	M@BTO NPs	HEK293T cell	Genetic engineering	Cancer therapy	[110]
Purine 18 with DSPE- PEG_2000_-NH_2_	mHAase@nP18	Baby hamster kidney (BHK-21) cell	Genetic engineering	Cancer therapy	[111]
Au nanocages (AuNs)	EpCam-RPAuNs	RBC	Lipid insertion	Cancer therapy	[112]
	PNMAuDIs	Neutrophil platelet	Membrane hybridization	Cancer therapy	[113]
	RBC-PL-robot	RBC platelet	Membrane hybridization	Bacteria therapy	[114]
Polypyrrole nanoparticles (PPy NPs)	PPy@ [R–P] NPs	RBC and PLT	Membrane hybridization	Cancer therapy	[115]
DSPE-PEOz	PEOz-liposome-dox	PLT	Lipid insertion	Cancer therapy	[116]
PLGA-ICG	PI@EPV	Cytomembrane vesicles (CMVs) and attenuated Salmonella outer membrane vesicles (OMVs)	Membrane hybridization	Cancer vaccines	[117]
Magnetic nanoparticle-loaded ICG	Fe_3_O_4_-ICG@IRM	ID8 ovarian cancer cell RBC	Membrane hybridization	Cancer therapy	[19]
Fe_3_O_4_@SiO_2_	HM- Fe_3_O_4_@SiO_2_/Tetra-DNA-Ag_2_S NDs	WBC and tumor cell	Membrane hybridization	Detection of Circulating tumor cells	[118]
Magnetic nanocluster (MNC)	RGD-M-MNC:siRNA	Macrophage	Lipid insertion	Gene therapy	[119]
	aAPC	Leukocyte	Metabolic glycan labeling	CTC detection	[120]
	JE-CM-MNs	Leukemia T lymphocyte	Genetic engineering	CTC detection	[121]
	Pa-M/Ti-NCs	Leukocyte	Metabolic glycan labeling	Cancer therapy	[122]
	A/M/C-MNC	Cancer cell	Metabolic glycan labeling	Cancer vaccines	[123]
	gCM-MN	Macrophages	Genetic engineering	Cancer therapy	[124]
	IOCMMNPs	Cancer cell	Chemical modification	Cancer therapy	[125]
	IOCMMNPs	HEK293 cells	Metabolic glycan labeling	Drug discovery	[126]
	HDFGFR4/CMMNPs	HEK293T cell	Genetic engineering	Drug discovery	[127]
Iron oxide magnetic nanoparticle (MN)	CSC-P-MN	PLT and cancer stem cell	Membrane hybridization	Cancer therapy	[128]
Mesoporous silica nanoparticles	CMSN-GOx	Cancer cell	Genetic engineering	Cancer therapy	[129]
	Ghodamine (green)-containing MSN-coated cells	Single cell	Direct chemical modification	Cell therapy	[130]
	DAazo@CMSN	NALM-6 cell	Lipid insertion	Leukemia therapy	[131]
	MSN@CM-GN_3_	RAW264.7 cells	Lipid insertion	Gene therapy	[24]
Magnesium-based core	Motor-toxoid	RBC	Lipid insertion	Vaccines	[132]
Superparamagnetic iron oxide nanoparticles (SPIONs)	USM[H]L	M2 macrophage-erythrocyte	Membrane hybridization	Inflammatory therapy	[20]
Meso-tetrakis (4-sulfonatophenyl) porphyrin (TPPS)	ANV	HEK 293T cell	Genetic engineering	Bacteria therapy	[133]
Membrane-penetrating helical polypeptide (P-Ben) and siSav1, a charge-reversal intermediate layer of poly (l-lysine)-cis-aconitic acid (PC)	BSPCA@HM NCs	PLT and macrophage	Membrane hybridization	Gene therapy	[36]
saALOX15-loaded mesoporous polydopamine (MPDA)	Ang-MMsaNPs	Macrophage	Genetic engineering	Cancer therapy	[134]
Oleic acid/ D-*α*-tocopherol polyethylene glycol succinate-lanthanide-doped nanoparticles loading GA and ICG	HMGINPs	Brain metastatic breast cancer cell and glioma cell	Membrane hybridization	Cancer therapy	[35]
Gboxin loaded poly (4-(4, 4, 5, 5-tetramethyl-1, 3, 2-dioxaborolan-2-yl) benzyl acrylate) (PEG-PHB)	HM-NPs@G	Cancer cell and mitochondrial	Membrane hybridization	Cancer therapy	[33]
Hyperbranched PEI25k loaded unmethylated cytosine-phosphate-guanine (CpG)	DBE@CCNPs	B16F10 cancer cell	Genetic engineering	Cancer vaccines	[135]
Melanin	Melanin@RBC-M	RBC/ MCF-7 cell	Membrane hybridization	Cancer therapy	[136]
Poly (benzobisthiadiazole-alt-thiophene) (pBBTT)	SPNE	Tumor cells and dendritic cells (DCs)	Membrane hybridization	Cancer therapy	[38]
Fibroin nanoparticles	MSNCs	Macrophage	Genetic engineering	Periodontal therapy	[137]
Poly (caprolactone)-ester endcap polymer (PCL)	PTX-PN@DiR-RV	RBC	Lipid insertion	Cancer therapy	[138]
Polydopamine (PDA)	PGT@cRGD-M	Osteosarcoma cell	Lipid insertion	Cancer therapy	[139]
Nanogel	PNG@mR&C	Bone mesenchymal stem cells	Genetic engineering	Osteoporosis therapy	[140]
(3E,7E)-3,7-bis (4-(2-decyltetradecyl)-4H-thieno [3,2-b] pyrrole-5,6-dione)-5,7-dihydro pyrrolo [2,3-f] indole-2,6 (1H,3H)-dione (BTPBF) as well as thienyl-diketopyrrolopyrrole (TDPP)	SPN-TF	293-FT cell	Genetic engineering	Cancer therapy	[141]
ZIF-8 MOF	CM-MOF-enzyme NP	RBC and macrophage	Membrane hybridization	Enzyme delivery	[142]
Porphyrin-based Zr-MOF (PCN-224)	PCN@FM	Tumor cells and DCs	Membrane hybridization	Cancer therapy	[143]
	NP@FM	Tumor cells and DCs	Membrane hybridization	Cancer vaccines	[144]

## Data Availability

Data are contained within the article.

## References

[1] Gavas S., Quazi S., Karpinski T.M. (2021). Nanoparticles for Cancer Therapy: Current Progress and Challenges. Nanoscale Res. Lett..

[2] Huang Y.Y., Ren J.S., Qu X.G. (2019). Nanozymes: Classification, Catalytic Mechanisms, Activity Regulation, and Applications. Chem. Rev..

[3] Fang R.H., Gao W.W., Zhang L.F. (2023). Targeting drugs to tumors using cell membrane-coated nanoparticles. Nat. Rev. Clin. Oncol..

[4] Mei H., Cai S.S., Huang D.N., Gao H.L., Cao J., He B. (2022). Carrier-free nanodrugs with efficient drug delivery and release for cancer therapy: From intrinsic physicochemical properties to external modification. Bioact. Mater..

[5] Huang L.L., Wu H.H., Xu D.H., Gao J.Q. (2022). Recent advances of cell membrane-derived biomimetic nanotechnology in cancer targeted drug delivery system. Acta Pharm. Sin. B.

[6] Suk J.S., Xu Q.G., Kim N., Hanes J., Ensign L.M. (2016). PEGylation as a strategy for improving nanoparticle-based drug and gene delivery. Adv. Drug Delivery Rev..

[7] Tian H.L., Zhang T.T., Qin S.Y., Huang Z., Zhou L., Shi J.Y., Nice E.C., Xie N., Huang C.H., Shen Z.S. (2022). Enhancing the therapeutic efficacy of nanoparticles for cancer treatment using versatile targeted strategies. J. Hematol. Oncol..

[8] Zhao H.L., Li N., Ma C.X., Wei Z.W., Zeng Q.Y., Zhang K.Y., Zhao N., Tang B.Z. (2023). An AIE probe for long-term plasma membrane imaging and membrane-targeted photodynamic therapy. Chin. Chem. Lett..

[9] Dai Q., Wilhelm S., Ding D., Syed A.M., Sindhwani S., Zhang Y.W., Chen Y.Y., MacMillan P., Chan W.C.W. (2018). Quantifying the Ligand-Coated Nanoparticle Delivery to Cancer Cells in Solid Tumors. ACS Nano.

[10] Hu C.-M.J., Zhang L., Aryal S., Cheung C., Fang R.H., Zhang L. (2011). Erythrocyte membrane-camouflaged polymeric nanoparticles as a biomimetic delivery platform. Proc. Natl. Acad. Sci. USA.

[11] Zhen X., Cheng P.H., Pu K.Y. (2019). Recent Advances in Cell Membrane-Camouflaged Nanoparticles for Cancer Phototherapy. Small.

[12] Yan Z., Wang D., Gao Y. (2023). Nanomaterials for the treatment of bacterial infection by photothermal/photodynamic synergism. Front. Bioeng. Biotechnol..

[13] Yan H.Z., Shao D., Lao Y.H., Li M.Q., Hu H.Z., Leong K.W. (2019). Engineering Cell Membrane-Based Nanotherapeutics to Target Inflammation. Adv. Sci..

[14] Hao H., Chen Y., Wu M. (2020). Biomimetic nanomedicine toward personalized disease theranostics. Nano Res..

[15] Lei W., Yang C., Wu Y., Ru G.Q., He X.L., Tong X.M., Wang S.B. (2022). Nanocarriers surface engineered with cell membranes for cancer targeted chemotherapy. J. Nanobiotechnol..

[16] Luk B.T., Zhang L.F. (2015). Cell membrane-camouflaged nanoparticles for drug delivery. J. Control. Release.

[17] Zhai Y.H., Su J.H., Ran W., Zhang P.C., Yin Q., Zhang Z.W., Yu H.J., Li Y.P. (2017). Preparation and Application of Cell Membrane-Camouflaged Nanoparticles for Cancer Therapy. Theranostics.

[18] Liang Y.J., Duan L., Lu J.P., Xia J. (2021). Engineering exosomes for targeted drug delivery. Theranostics.

[19] Xiong J.Q., Wu M., Chen J.L., Liu Y.F., Chen Y.R., Fan G.L., Liu Y.Y., Cheng J., Wang Z.H., Wang S.X. (2021). Cancer-Erythrocyte Hybrid Membrane-Camouflaged Magnetic Nanoparticles with Enhanced Photothermal-Immunotherapy for Ovarian Cancer. ACS Nano.

[20] Chen R., Yang J., Wu M., Zhao D., Yuan Z., Zeng L., Hu J., Zhang X., Wang T., Xu J. (2023). M2 Macrophage Hybrid Membrane-Camouflaged Targeted Biomimetic Nanosomes to Reprogram Inflammatory Microenvironment for Enhanced Enzyme-Thermo-Immunotherapy. Adv. Mater..

[21] Dehaini D., Wei X., Fang R.H., Masson S., Angsantikul P., Luk B.T., Zhang Y., Ying M., Jiang Y., Kroll A.V. (2017). Erythrocyte-Platelet Hybrid Membrane Coating for Enhanced Nanoparticle Functionalization. Adv. Mater..

[22] Wang Q., Cheng H., Peng H.S., Zhou H., Li P.Y., Langer R. (2015). Non-genetic engineering of cells for drug delivery and cell-based therapy. Adv. Drug Delivery Rev..

[23] Ai X.Z., Wang S.Y., Duan Y.O., Zhang Q.Z., Chen M.S., Gao W.W., Zhang L.F. (2021). Emerging Approaches to Functionalizing Cell Membrane-Coated Nanoparticles. Biochemistry.

[24] He X., Chang Z., Chen F., Zhang W., Sun M., Shi T., Liu J., Chen P., Zhang K., Guan S. (2024). Engineering a biomimetic system for hepatocyte-specific RNAi treatment of non-alcoholic fatty liver disease. Acta Biomater..

[25] Chai Z.L., Ran D.N., Lu L.W., Zhan C.Y., Ruan H.T., Hu X.F., Xie C., Jiang K., Li J.Y., Zhou J.F. (2019). Ligand-Modified Cell Membrane Enables the Targeted Delivery of Drug Nanocrystals to Glioma. ACS Nano.

[26] Weise K., Huster D., Kapoor S., Triola G., Waldmann H., Winter R. (2013). Gibbs energy determinants of lipoprotein insertion into lipid membranes: The case study of Ras proteins. Faraday Discuss..

[27] Shi P., Wang X.L., Davis B., Coyne J., Dong C., Reynolds J., Wang Y. (2020). In Situ Synthesis of an Aptamer-Based Polyvalent Antibody Mimic on the Cell Surface for Enhanced Interactions between Immune and Cancer Cells. Angew. Chem. Int. Ed..

[28] Sui S.F., Wu H., Guo Y., Chen K.S. (1994). Conformational-Changes of Melittin Upon Insertion into Phospholipid Monolayer and Vesicle. J. Biochem..

[29] Zhang Q., Zhou J., Zhou J., Fang R.H., Gao W., Zhang L. (2021). Lure-and-kill macrophage nanoparticles alleviate the severity of experimental acute pancreatitis. Nat. Commun..

[30] Fang R.N.H., Hu C.M.J., Chen K.N.H., Luk B.T., Carpenter C.W., Gao W.W., Li S.L., Zhang D.E., Lu W.Y., Zhang L.F. (2013). Lipid-insertion enables targeting functionalization of erythrocyte membrane-cloaked nanoparticles. Nanoscale.

[31] Kato K., Itoh C., Yasukouchi T., Nagamune T. (2004). Rapid protein anchoring into the membranes of mammalian cells using oleyl chain and poly(ethylene glycol) derivatives. Biotechnol. Prog..

[32] Song W.L., Jia P.F., Ren Y.P., Xue J.M., Zhou B.Q., Xu X.K., Shan Y.S., Deng J., Zhou Q.H. (2023). Engineering white blood cell membrane-camouflaged nanocarriers for inflammation-related therapeutics. Bioact. Mater..

[33] Zou Y., Sun Y.J., Wang Y.B., Zhang D.Y., Yang H.Q., Wang X., Zheng M., Shi B.Y. (2023). Cancer cell-mitochondria hybrid membrane coated Gboxin loaded nanomedicines for glioblastoma treatment. Nat. Commun..

[34] Chen H.Y., Deng J., Wang Y., Wu C.Q., Li X., Dai H.W. (2020). Hybrid cell membrane-coated nanoparticles: A multifunctional biomimetic platform for cancer diagnosis and therapy. Acta Biomater..

[35] Chi S., Zhang L., Cheng H., Chang Y., Zhao Y., Wang X., Liu Z. (2023). Biomimetic Nanocomposites Camouflaged with Hybrid Cell Membranes for Accurate Therapy of Early-Stage Glioma. Angew. Chem. Int. Ed..

[36] Zhou Y., Liang Q.J., Wu X.J., Duan S.Z., Ge C.L., Ye H., Lu J.H., Zhu R.Y., Chen Y.B., Meng F.H. (2023). siRNA Delivery against Myocardial Ischemia Reperfusion Injury Mediated by Reversibly Camouflaged Biomimetic Nanocomplexes. Adv. Mater..

[37] Anguille S., Smits E.L., Lion E., van Tendeloo V.F., Berneman Z.N. (2014). Clinical use of dendritic cells for cancer therapy. Lancet Oncol..

[38] Xu C., Jiang Y., Han Y., Pu K., Zhang R. (2021). A Polymer Multicellular Nanoengager for Synergistic NIR-II Photothermal Immunotherapy. Adv. Mater..

[39] Liu W.S., Wu L.L., Chen C.M., Zheng H., Gao J., Lu Z.M., Li M. (2023). Lipid-hybrid cell-derived biomimetic functional materials: A state-of-the-art multifunctional weapon against tumors. Mater. Today Bio.

[40] Sletten E.M., Bertozzi C.R. (2009). Bioorthogonal Chemistry: Fishing for Selectivity in a Sea of Functionality. Angew. Chem. Int. Ed..

[41] Grupi A., Ashur I., Degani-Katzav N., Yudovich S., Shapira Z., Marzouq A., Morgenstein L., Mandel Y., Weiss S. (2019). Interfacing the Cell with “Biomimetic Membrane Proteins”. Small.

[42] Cai F.Y., Ren Y.F., Dai J.J., Yang J.M., Shi X.A. (2023). Effects of Various Cell Surface Engineering Reactions on the Biological Behavior of Mammalian Cells. Macromol. Biosci..

[43] Xi Z., Kong H., Chen Y., Deng J., Xu W., Liang Y., Zhang Y. (2022). Metal- and Strain-Free Bioorthogonal Cycloaddition of o-Diones with Furan-2(3H)-one as Anionic Cycloaddend. Angew. Chem. Int. Ed..

[44] Lutterotti A., Yousef S., Sputtek A., Stürner K.H., Stellmann J.P., Breiden P., Reinhardt S., Schulze C., Bester M., Heesen C. (2013). Antigen-Specific Tolerance by Autologous Myelin Peptide-Coupled Cells: A Phase 1 Trial in Multiple Sclerosis. Sci. Transl. Med..

[45] Jones R.B., Mueller S., Kumari S., Vrbanac V., Genel S., Tager A.M., Allen T.M., Walker B.D., Irvine D.J. (2017). Antigen recognition-triggered drug delivery mediated by nanocapsule-functionalized cytotoxic T-cells. Biomaterials.

[46] Wang W.S., Gan Q., Zhang Y.Q., Lu X., Wang H.X., Zhang Y.K., Hu H., Chen L.N., Shi L.X., Wang S.T. (2021). Polymer-Assisted Metallization of Mammalian Cells. Adv. Mater..

[47] Sun Q., Liu G.Q., Wu H.B., Xue H.D., Zhao Y., Wang Z.L., Wei Y., Wang Z.M., Tao L. (2017). Fluorescent Cell-Conjugation by a Multifunctional Polymer: A New Application of the Hantzsch Reaction. ACS Macro Lett..

[48] Cheng Q.Z., Kang Y., Yao B., Dong J.R., Zhu Y.L., He Y.L., Ji X.Y. (2023). Genetically Engineered-Cell-Membrane Nanovesicles for Cancer Immunotherapy. Adv. Sci..

[49] Smith B.A.H., Bertozzi C.R. (2021). The clinical impact of glycobiology: Targeting selectins, Siglecs and mammalian glycans. Nat. Rev. Drug Discov..

[50] Nie W., Wu G., Zhang J., Huang L.-L., Ding J., Jiang A., Zhang Y., Liu Y., Li J., Pu K. (2020). Responsive Exosome Nano-bioconjugates for Synergistic Cancer Therapy. Angew. Chem. Int. Ed..

[51] Han S.S., Lee D.E., Shim H.E., Lee S., Jung T., Oh J.H., Lee H.A., Moon S.H., Jeon J., Yoon S. (2017). Physiological Effects of Ac_4_ManNAz and Optimization of Metabolic Labeling for Cell Tracking. Theranostics.

[52] Liu Z., Sun M., Zhang W., Ren J., Qu X. (2023). Target-Specific Bioorthogonal Reactions for Precise Biomedical Applications. Angew. Chem. Int. Ed..

[53] Meng X.Z., Wang J.J., Zhou J.D., Tian Q.M., Qie B., Zhou G., Duan W., Zhu Y.M. (2021). Tumor cell membrane-based peptide delivery system targeting the tumor microenvironment for cancer immunotherapy and diagnosis. Acta Biomater..

[54] Meng D.D., Pan H., He W., Jiang X., Liang Z.G., Zhang X., Xu X.Y., Wang Z.X., Zheng J.L., Gong P. (2022). In Situ Activated NK Cell as Bio-Orthogonal Targeted Live-Cell Nanocarrier Augmented Solid Tumor Immunotherapy. Adv. Funct. Mater..

[55] Au K.M., Tisch R., Wang A.Z. (2021). Bioengineering of Beta Cells with Immune Checkpoint Ligand as a Treatment for Early-Onset Type 1 Diabetes Mellitus. ACS Nano.

[56] Zhao T., Masuda T., Takai M. (2021). pH-Responsive Water-Soluble Polymer Carriers for Cell-Selective Metabolic Sialylation Labeling. Anal. Chem..

[57] Cheng B., Tang Q., Zhang C., Chen X. (2021). Glycan Labeling and Analysis in Cells and In Vivo. Annu. Rev. Anal. Chem..

[58] Xie R., Hong S.L., Chen X. (2013). Cell-selective metabolic labeling of biomolecules with bioorthogonal functionalities. Curr. Opin. Chem. Biol..

[59] Cheng B., Xie R., Dong L., Chen X. (2016). Metabolic Remodeling of Cell-Surface Sialic Acids: Principles, Applications, and Recent Advances. Chembiochem.

[60] Wang D., Jiang S., Zhang F., Ma S., Heng B.C., Wang Y., Zhu J., Xu M., He Y., Wei Y. (2021). Cell Membrane Vesicles with Enriched CXCR4 Display Enhances Their Targeted Delivery as Drug Carriers to Inflammatory Sites. Adv. Sci..

[61] Kershaw M.H., Westwood J.A., Darcy P.K. (2013). Gene-engineered T cells for cancer therapy. Nat. Rev. Cancer.

[62] Hu Y.C., Cao Z.H., Zheng L.G., Shen J.T., Zhao W., Dai L. (2023). Applications of CRISPR-Cas Technologies in Microbiome Engineering. Chem. J. Chin. Univ..

[63] Xiao H., Li Y.K., Xing X.W. (2023). Recent Advances in Chemical Control of CRISPR/Cas9 Genome Editing Technology. Chem. J. Chin. Univ..

[64] Yang Z.L., Zhang Z.S. (2018). Engineering strategies for enhanced production of protein and bio-products in: A review. Biotechnol. Adv..

[65] Milone M.C., O’Doherty U. (2018). Clinical use of lentiviral vectors. Leukemia.

[66] Abbina S., Siren E.M.J., Moon H., Kizhakkedathu J.N. (2017). Surface Engineering for Cell-Based Therapies: Techniques for Manipulating Mammalian Cell Surfaces. ACS Biomater. Sci. Eng..

[67] Zhou W.Q., Guo S.C., Liu M.L., Burow M.E., Wang G.D. (2019). Targeting CXCL12/CXCR4 Axis in Tumor Immunotherapy. Curr. Med. Chem..

[68] Ma J.N., Zhang S.Q., Liu J., Liu F.Y., Du F., Li M., Chen A.T., Bao Y.M., Suh H.W., Avery J. (2019). Targeted Drug Delivery to Stroke via Chemotactic Recruitment of Nanoparticles Coated with Membrane of Engineered Neural Stem Cells. Small.

[69] Liu J.Q., Sun Y.T., Zeng X.H., Liu Y., Liu C.Z., Zhou Y., Liu Y.G., Sun G.H., Guo M.X. (2023). Engineering and Characterization of an Artificial Drug-Carrying Vesicles Nanoplatform for Enhanced Specifically Targeted Therapy of Glioblastoma. Adv. Mater..

[70] Feng M.Y., Xiong G.B., Cao Z., Yang G., Zheng S.L., Song X.J., You L., Zheng L.F., Zhang T.P., Zhao Y.P. (2017). PD-1/PD-L1 and immunotherapy for pancreatic cancer. Cancer Lett..

[71] Zhang X., Wang C., Wang J., Hu Q., Langworthy B., Ye Y., Sun W., Lin J., Wang T., Fine J. (2018). PD-1 Blockade Cellular Vesicles for Cancer Immunotherapy. Adv. Mater..

[72] Li B., Yang T., Liu J., Yu X.X., Li X.Y., Qin F., Zheng J.F., Liang J.X., Zeng Y.Y., Zhou Z.H. (2023). Genetically engineered PD-1 displaying nanovesicles for synergistic checkpoint blockades and chemo-metabolic therapy against non-small cell lung cancer. Acta Biomater..

[73] Krishnan N., Peng F.-X., Mohapatra A., Fang R.H., Zhang L. (2023). Genetically engineered cellular nanoparticles for biomedical applications. Biomaterials.

[74] Harish V., Tewari D., Gaur M., Yadav A.B., Swaroop S., Bechelany M., Barhoum A. (2022). Review on Nanoparticles and Nanostructured Materials: Bioimaging, Biosensing, Drug Delivery, Tissue Engineering, Antimicrobial, and Agro-Food Applications. Nanomaterials.

[75] Rao L., Meng Q.-F., Bu L.-L., Cai B., Huang Q., Sun Z.-J., Zhang W.-F., Li A., Guo S.-S., Liu W. (2017). Erythrocyte Membrane-Coated Upconversion Nanoparticles with Minimal Protein Adsorption for Enhanced Tumor Imaging. ACS Appl. Mater. Interfaces.

[76] Li M., Fang H., Liu Q., Gai Y., Yuan L., Wang S., Li H., Hou Y., Gao M., Lan X. (2020). Red blood cell membrane-coated upconversion nanoparticles for pretargeted multimodality imaging of triple-negative breast cancer. Biomater. Sci..

[77] Wang Y., Qiu Y., Chen S., Huang J., Hu X., Chen J., Wang S., Yang X., Zhang Y., Zhu Y. (2023). Functionalized Tumor Cell Membrane-Camouflaged Photo-Activatable Nanoparticle for Spatiotemporal Antitumor Therapy. Chem. Eng. J..

[78] Deng J., Xu S., Hu W., Xun X., Zheng L., Su M. (2018). Tumor targeted, stealthy and degradable bismuth nanoparticles for enhanced X-ray radiation therapy of breast cancer. Biomaterials.

[79] Zou Y., Liu Y., Yang Z., Zhang D., Lu Y., Zheng M., Xue X., Geng J., Chung R., Shi B. (2023). Effective and Targeted Human Orthotopic Glioblastoma Xenograft Therapy via a Multifunctional Biomimetic Nanomedicine (vol 30, 1803717, 2018). Adv. Mater..

[80] Wang Y.C., Luan Z.Y., Zhao C.Y., Bai C.H., Yang K.J. (2020). Target delivery selective CSF-1R inhibitor to tumor-associated macrophages erythrocyte-cancer cell hybrid membrane camouflaged pH-responsive copolymer micelle for cancer immunotherapy. Eur. J. Pharm. Sci..

[81] Xu J.P., Wang X.Q., Yin H.Y., Cao X., Hu Q.Y., Lv W., Xu Q.W., Gu Z., Xin H.L. (2019). Sequentially Site-Specific Delivery of Thrombolytics and Neuroprotectant for Enhanced Treatment of Ischemic Stroke. ACS Nano.

[82] Park J.H., Mohapatra A., Zhou J., Holay M., Krishnan N., Gao W., Fang R.H., Zhang L. (2021). Virus-Mimicking Cell Membrane-Coated Nanoparticles for Cytosolic Delivery of mRNA. Angew. Chem. Int. Ed..

[83] Guo Y., Wang D., Song Q., Wu T., Zhuang X., Bao Y., Kong M., Qj Y., Tan S., Zhang Z. (2015). Erythrocyte Membrane-Enveloped Polymeric Nanoparticles as Nanovaccine for Induction of Antitumor Immunity against Melanoma. ACS Nano.

[84] Zhang Z., Qian H., Huang J., Sha H., Zhang H., Yu L., Liu B., Hua D., Qian X. (2018). Anti-EGFR-iRGD recombinant protein modified biomimetic nanoparticles loaded with gambogic acid to enhance targeting and antitumor ability in colorectal cancer treatment. Int. J. Nanomed..

[85] Hu Q.Y., Sun W.J., Qian C.G., Wang C., Bomba H., Gu Z. (2016). Anticancer platelet-mimicking nanovehicles. Abstr. Pap. Am. Chem. Soc..

[86] Liu Z.M., Zhou X.F., Li Q., Shen Y.Q., Zhou T.H., Liu X.R. (2023). Macrophage-evading and tumor-specific apoptosis inducing nanoparticles for targeted cancer therapy. Acta. Pharm. Sin. B.

[87] Shen S.F., Dai H.X., Fei Z.Y., Chai Y., Hao Y., Fan Q., Dong Z.L., Zhu Y.J., Xu J.L., Ma Q.L. (2021). Immunosuppressive Nanoparticles for Management of Immune-Related Adverse Events in Liver. ACS Nano.

[88] Park J.H., Jiang Y., Zhou J., Gong H., Mohapatra A., Heo J., Gao W., Fang R.H., Zhang L. (2021). Genetically engineered cell membrane-coated nanoparticles for targeted delivery of dexamethasone to inflamed lungs. Sci. Adv..

[89] Krishnan N., Jiang Y., Zhou J., Mohapatra A., Peng F.-X., Duan Y., Holay M., Chekuri S., Guo Z., Gao W. (2023). A modular approach to enhancing cell membrane-coated nanoparticle functionality using genetic engineering. Nat. Nanotechnol..

[90] Chen Z., Zhao P., Luo Z., Zheng M., Tian H., Gong P., Gao G., Pan H., Liu L., Ma A. (2016). Cancer Cell Membrane–Biomimetic Nanoparticles for Homologous-Targeting Dual-Modal Imaging and Photothermal Therapy. ACS Nano.

[91] Han Y., Pan H., Li W., Chen Z., Ma A., Yin T., Liang R., Chen F., Ma Y., Jin Y. (2019). T Cell Membrane Mimicking Nanoparticles with Bioorthogonal Targeting and Immune Recognition for Enhanced Photothermal Therapy. Adv. Sci..

[92] Ai X., Wang D., Honko A., Duan Y., Gavrish I., Fang R.H., Griffiths A., Gao W., Zhang L. (2021). Surface Glycan Modification of Cellular Nanosponges to Promote SARS-CoV-2 Inhibition. J. Am. Chem. Soc..

[93] Yin T., Fan Q., Hu F., Ma X., Yin Y., Wang B., Kuang L., Hu X., Xu B., Wang Y. (2022). Engineered Macrophage-Membrane-Coated Nanoparticles with Enhanced PD-1 Expression Induce Immunomodulation for a Synergistic and Targeted Antiglioblastoma Activity. Nano Lett..

[94] Yang R., Xu J., Xu L., Sun X., Chen Q., Zhao Y., Peng R., Liu Z. (2018). Cancer Cell Membrane-Coated Adjuvant Nanoparticles with Mannose Modification for Effective Anticancer Vaccination. ACS Nano.

[95] Fu S., Liang M., Wang Y., Cui L., Gao C., Chu X., Liu Q., Feng Y., Gong W., Yang M. (2019). Dual-Modified Novel Biomimetic Nanocarriers Improve Targeting and Therapeutic Efficacy in Glioma. ACS Appl. Mater. Interfaces.

[96] Zhao H.J., Zhao B.B., Li L., Ding K.L., Xiao H.F., Zheng C.X., Sun L.L., Zhang Z.Z., Wang L. (2020). Biomimetic Decoy Inhibits Tumor Growth and Lung Metastasis by Reversing the Drawbacks of Sonodynamic Therapy. Adv. Healthc. Mater..

[97] Kim H.Y., Kang M., Choo Y.W., Go S.H., Kwon S.P., Song S.Y., Sohn H.S., Hong J., Kim B.S. (2019). Immunomodulatory Lipocomplex Functionalized with Photosensitizer-Embedded Cancer Cell Membrane Inhibits Tumor Growth and Metastasis. Nano Lett..

[98] Fan Y.Y., Cui Y.X., Hao W.Y., Chen M.Y., Liu Q.Q., Wang Y.L., Yang M.Y., Li Z.P., Gong W., Song S.Y. (2021). Carrier-free highly drug-loaded biomimetic nanosuspensions encapsulated by cancer cell membrane based on homology and active targeting for the treatment of glioma. Bioact. Mater..

[99] Ding L., Zhang X., Yu P., Peng F., Sun Y., Wu Y., Luo Z., Li H., Zeng Y., Wu M. (2023). Genetically engineered nanovesicles mobilize synergistic antitumor immunity by ADAR1 silence and PDL1 blockade. Mol. Ther..

[100] Lv W., Xu J., Wang X., Li X., Xu Q., Xin H. (2018). Bioengineered Boronic Ester Modified Dextran Polymer Nanoparticles as Reactive Oxygen Species Responsive Nanocarrier for Ischemic Stroke Treatment. ACS Nano.

[101] Chai Z., Hu X., Wei X., Zhan C., Lu L., Jiang K., Su B., Ruan H., Ran D., Fang R.H. (2017). A facile approach to functionalizing cell membrane-coated nanoparticles with neurotoxin-derived peptide for brain-targeted drug delivery. J. Control. Release.

[102] Huang D., Wang Q., Cao Y., Yang H., Li M., Wu F., Zhang Y., Chen G., Wang Q. (2023). Multiscale NIR-II Imaging-Guided Brain-Targeted Drug Delivery Using Engineered Cell Membrane Nanoformulation for Alzheimer’s Disease Therapy. ACS Nano.

[103] Xu Y., Du L., Han B., Wang Y., Fei J., Xia K., Zhai Y., Yu Z. (2023). Black phosphorus quantum dots camouflaged with platelet-osteosarcoma hybrid membrane and doxorubicin for combined therapy of osteosarcoma. J. Nanobiotechnol..

[104] Liu W., Ruan M.L., Liu L.M., Ji X., Ma Y.D., Yuan P.F., Tang G.H., Lin H.S., Dai J., Xue W. (2020). Self-activated therapeutic cascade of erythrocyte membrane-cloaked iron-mineralized enzymes. Theranostics.

[105] Duan Y., Wu M., Hu D., Pan Y., Hu F., Liu X., Thakor N., Ng W.H., Liu X., Sheng Z. (2020). Biomimetic Nanocomposites Cloaked with Bioorthogonally Labeled Glioblastoma Cell Membrane for Targeted Multimodal Imaging of Brain Tumors. Adv. Funct. Mater..

[106] Rao L., Meng Q.-F., Huang Q., Wang Z., Yu G.-T., Li A., Ma W., Zhang N., Guo S.-S., Zhao X.-Z. (2018). Platelet-Leukocyte Hybrid Membrane-Coated Immunomagnetic Beads for Highly Efficient and Highly Specific Isolation of Circulating Tumor Cells. Adv. Funct. Mater..

[107] Guliz A.K., Sanlier S.H. (2020). Erythrocyte membrane vesicles coated biomimetic and targeted doxorubicin nanocarrier: Development, characterization and in vitro studies. J. Mol. Struct..

[108] Zhan Z., Zeng W.Q., Liu J.Z., Zhang L., Cao Y., Li P., Ran H.T., Wang Z.G. (2023). Engineered Biomimetic Copper Sulfide Nanozyme Mediates “Don’t Eat Me” Signaling for Photothermal and Chemodynamic Precision Therapies of Breast Cancer. ACS Appl. Mater. Interfaces.

[109] Wang D., Dong H., Li M., Cao Y., Yang F., Zhang K., Dai W., Wang C., Zhang X. (2018). Erythrocyte–Cancer Hybrid Membrane Camouflaged Hollow Copper Sulfide Nanoparticles for Prolonged Circulation Life and Homotypic-Targeting Photothermal/Chemotherapy of Melanoma. ACS Nano.

[110] Tang Q., Sun S., Wang P., Sun L., Wang Y., Zhang L., Xu M., Chen J., Wu R., Zhang J. (2023). Genetically Engineering Cell Membrane-Coated BTO Nanoparticles for MMP2-Activated Piezocatalysis-Immunotherapy. Adv. Mater..

[111] Xu S., Shi X., Ren E., Zhang J., Gao X., Mu D., Liu C., Liu G. (2022). Genetically Engineered Nanohyaluronidase Vesicles: A Smart Sonotheranostic Platform for Enhancing Cargo Penetration of Solid Tumors. Adv. Funct. Mater..

[112] Zhu D.-M., Xie W., Xiao Y.-S., Suo M., Zan M.-H., Liao Q.-Q., Hu X.-J., Chen L.-B., Chen B., Wu W.-T. (2018). Erythrocyte membrane-coated gold nanocages for targeted photothermal and chemical cancer therapy. Nanotechnology.

[113] Ye H., Wang K.Y., Lu Q., Zhao J., Wang M.L., Kan Q.M., Zhang H.T., Wang Y.J., He Z.G., Sun J. (2020). Nanosponges of circulating tumor-derived exosomes for breast cancer metastasis inhibition. Biomaterials.

[114] De Avila B.E.F., Angsantikul P., Ramírez-Herrera D.E., Soto F., Teymourian H., Dehaini D., Chen Y.J., Zhang L.F., Wang J. (2018). Hybrid biomembrane-functionalized nanorobots for concurrent removal of pathogenic bacteria and toxins. Sci. Robot..

[115] Liu Y., Wang X.J., Ouyang B.S., Liu X.P., Du Y., Cai X.Z., Guo H.S., Pang Z.Q., Yang W.L., Shen S. (2018). Erythrocyte-platelet hybrid membranes coating polypyrrol nanoparticles for enhanced delivery and photothermal therapy. J. Mater. Chem. B.

[116] Liu G.N., Zhao X., Zhang Y.L., Xu J.C., Xu J.Q., Li Y., Min H., Shi J., Zhao Y., Wei J.Y. (2019). Engineering Biomimetic Platesomes for pH-Responsive Drug Delivery and Enhanced Antitumor Activity. Adv. Mater..

[117] Chen Q., Huang G.J., Wu W.T., Wang J.W., Hu J.W., Mao J.M., Chu P.K., Bai H.Z., Tang G.P. (2020). A Hybrid Eukaryotic-Prokaryotic Nanoplatform with Photothermal Modality for Enhanced Antitumor Vaccination. Adv. Mater..

[118] Ding C.P., Zhang C.L., Cheng S.S., Xian Y.Z. (2020). Multivalent Aptamer Functionalized AgS Nanodots/Hybrid Cell Membrane-Coated Magnetic Nanobioprobe for the Ultrasensitive Isolation and Detection of Circulating Tumor Cells. Adv. Funct. Mater..

[119] Zhang F., Zhao L., Wang S., Yang J., Lu G., Luo N., Gao X., Ma G., Xie H.-Y., Wei W. (2018). Construction of a Biomimetic Magnetosome and Its Application as a SiRNA Carrier for High-Performance Anticancer Therapy. Adv. Funct. Mater..

[120] Zhang Q., Wei W., Wang P., Zuo L., Li F., Xu J., Xi X., Gao X., Ma G., Xie H.-Y. (2017). Biomimetic Magnetosomes as Versatile Artificial Antigen-Presenting Cells to Potentiate T-Cell-Based Anticancer Therapy. ACS Nano.

[121] Jiang X., Zhang X., Guo C., Ma B., Liu Z., Du Y., Wang B., Li N., Huang X., Ou L. (2023). Genetically Engineered Cell Membrane-Coated Magnetic Nanoparticles for High-Performance Isolation of Circulating Tumor Cells. Adv. Funct. Mater..

[122] Zhang F., Li F., Lu G.-H., Nie W., Zhang L., Lv Y., Bao W., Gao X., Wei W., Pu K. (2019). Engineering Magnetosomes for Ferroptosis/Immunomodulation Synergism in Cancer. ACS Nano.

[123] Li F., Nie W., Zhang F., Lu G., Lv C., Lv Y., Bao W., Zhang L., Wang S., Gao X. (2019). Engineering Magnetosomes for High-Performance Cancer Vaccination. ACS Cent. Sci..

[124] Rao L., Zhao S.K., Wen C., Tian R., Lin L., Cai B., Sun Y., Kang F., Yang Z., He L. (2020). Activating Macrophage-Mediated Cancer Immunotherapy by Genetically Edited Nanoparticles. Adv. Mater..

[125] Bu Y., Zhang X., Zhu A., Li L., Xie X., Wang S. (2021). Inside-Out-Oriented Cell Membrane Biomimetic Magnetic Nanoparticles for High-Performance Drug Lead Discovery. Anal. Chem..

[126] Zhang X., Zhen X., Yang Y., Feng Q., Yuan W., Xie X. (2023). Precise assembly of inside-out cell membrane camouflaged nanoparticles via bioorthogonal reactions for improving drug leads capturing. Acta. Pharm. Sin. B.

[127] Bu Y., Wu D., Zhao Y., Wang G., Dang X., Xie X., Wang S. (2023). Genetically Engineered Cell Membrane-Coated Nanoparticles with High-Density Customized Membrane Receptor for High-Performance Drug Lead Discovery. ACS Appl. Mater. Interfaces.

[128] Bu L.-L., Rao L., Yu G.-T., Chen L., Deng W.-W., Liu J.-F., Wu H., Meng Q.-F., Guo S.-S., Zhao X.-Z. (2019). Cancer Stem Cell-Platelet Hybrid Membrane-Coated Magnetic Nanoparticles for Enhanced Photothermal Therapy of Head and Neck Squamous Cell Carcinoma. Adv. Funct. Mater..

[129] Xie W., Deng W.W., Zan M.H., Rao L., Yu G.T., Zhu D.M., Wu W.T., Chen B., Ji L.W., Chen L.B. (2019). Cancer Cell Membrane Camouflaged Nanoparticles to Realize Starvation Therapy Together with Checkpoint Blockades for Enhancing Cancer Therapy. ACS Nano.

[130] Kim H., Shin K., Park O.K., Choi D., Kim H.D., Baik S., Lee S.H., Kwon S.H., Yarema K.J., Hong J. (2018). General and Facile Coating of Single Cells via Mild Reduction. J. Am. Chem. Soc..

[131] Dong X., Mu L.-L., Liu X.-L., Zhu H., Yang S.-C., Lai X., Liu H.-J., Feng H.-Y., Lu Q., Zhou B.-B.S. (2020). Biomimetic, Hypoxia-Responsive Nanoparticles Overcome Residual Chemoresistant Leukemic Cells with Co-Targeting of Therapy-Induced Bone Marrow Niches. Adv. Funct. Mater..

[132] Wei X.L., Beltrán-Gastélum M., Karshalev E., de Avila B.E.F., Zhou J.R., Ran D.N., Angsantikul P., Fang R.H., Wang J., Zhang L.F. (2019). Biomimetic Micromotor Enables Active Delivery of Antigens for Oral Vaccination. Nano Lett..

[133] Pang X., Liu X., Cheng Y., Zhang C., Ren E., Liu C., Zhang Y., Zhu J., Chen X.Y., Liu G. (2019). Sono-Immunotherapeutic Nanocapturer to Combat Multidrug-Resistant Bacterial Infections. Adv. Mater..

[134] Cao Z., Liu X., Zhang W., Zhang K., Pan L., Zhu M., Qin H., Zou C., Wang W., Zhang C. (2023). Biomimetic Macrophage Membrane-Camouflaged Nanoparticles Induce Ferroptosis by Promoting Mitochondrial Damage in Glioblastoma. ACS Nano.

[135] Liu S., Wu J., Feng Y., Guo X., Li T., Meng M., Chen J., Chen D., Tian H. (2023). CD47KO/CRT dual-bioengineered cell membrane-coated nanovaccine combined with anti-PD-L1 antibody for boosting tumor immunotherapy. Bioact. Mater..

[136] Jiang Q., Liu Y., Guo R., Yao X., Sung S., Pang Z., Yang W. (2019). Erythrocyte-cancer hybrid membrane-camouflaged melanin nanoparticles for enhancing photothermal therapy efficacy in tumors. Biomaterials.

[137] Deng Y., Ren M., He P., Liu F., Wang X., Zhou C., Li Y., Yang S. (2023). Genetically engineered cell membrane-coated nanoparticles for antibacterial and immunoregulatory dual-function treatment of ligature-induced periodontitis. Front. Bioeng. Biotechnol..

[138] Su J., Sun H., Meng Q., Yin Q., Zhang P., Zhang Z., Yu H., Li Y. (2016). Bioinspired Nanoparticles with NIR-Controlled Drug Release for Synergetic Chemophotothermal Therapy of Metastatic Breast Cancer. Adv. Funct. Mater..

[139] Guo H., Zhang W., Wang L., Shao Z., Huang X. (2022). Biomimetic cell membrane-coated glucose/oxygen-exhausting nanoreactor for remodeling tumor microenvironment in targeted hypoxic tumor therapy. Biomaterials.

[140] Cui Y., Lv B., Li Z., Ma C., Gui Z., Geng Y., Liu G., Sang L., Xu C., Min Q. (2023). Bone-Targeted Biomimetic Nanogels Re-Establish Osteoblast/Osteoclast Balance to Treat Postmenopausal Osteoporosis. Small.

[141] Wei Z., Xin F., Yang S., Zhang C., Wang B., Xue F., Guo Z. (2022). Genetically Engineered Cell Membrane Modified Conjugated Polymer Nanoparticles for NIR-II Photothermal Therapy. Adv. Mater. Interfaces.

[142] Wang S.Y., Kai M.X., Duan Y.O., Zhou Z.D., Fang R.H., Gao W.W., Zhang L.F. (2022). Membrane Cholesterol Depletion Enhances Enzymatic Activity of Cell-Membrane-Coated Metal-Organic-Framework Nanoparticles. Angew. Chem. Int. Ed..

[143] Liu W.L., Zou M.Z., Liu T., Zeng J.Y., Li X., Yu W.Y., Li C.X., Ye J.J., Song W., Feng J. (2019). Expandable Immunotherapeutic Nanoplatforms Engineered from Cytomembranes of Hybrid Cells Derived from Cancer and Dendritic Cells. Adv. Mater..

[144] Liu W.-L., Zou M.-Z., Liu T., Zeng J.-Y., Li X., Yu W.-Y., Li C.-X., Ye J.-J., Song W., Feng J. (2019). Cytomembrane nanovaccines show therapeutic effects by mimicking tumor cells and antigen presenting cells. Nat. Commun..

[145] Ge J.P., Hu Y.X., Biasini M., Beyermann W.P., Yin Y.D. (2007). Superparamagnetic magnetite colloidal nanocrystal clusters. Angew. Chem. Int. Edit.

[146] Rao L., Bu L.L., Cai B., Xu J.H., Li A., Zhang W.F., Sun Z.J., Guo S.S., Liu W., Wang T.H. (2016). Cancer Cell Membrane-Coated Upconversion Nanoprobes for Highly Specific Tumor Imaging. Adv. Mater..

[147] Liu Y.Y., Meng X.F., Bu W.B. (2019). Upconversion-based photodynamic cancer therapy. Coord. Chem. Rev..

[148] Zeng J.-Y., Zou M.-Z., Zhang M., Wang X.-S., Zeng X., Cong H., Zhang X.-Z. (2018). π-Extended Benzoporphyrin-Based Metal–Organic Framework for Inhibition of Tumor Metastasis. ACS Nano.

[149] Zheng Q.Y., Liu X.M., Zheng Y.F., Yeung K.W.K., Cui Z.D., Liang Y.Q., Li Z.Y., Zhu S.L., Wang X.B., Wu S.L. (2021). The recent progress on metal-organic frameworks for phototherapy. Chem. Soc. Rev..

[150] Kong F.Y., Zhang J.W., Li R.F., Wang Z.X., Wang W.J., Wang W. (2017). Unique Roles of Gold Nanoparticles in Drug Delivery, Targeting and Imaging Applications. Molecules.

[151] Han S.T., Hu L., Wang X., Zhou Y., Zeng Y.J., Ruan S., Pan C., Peng Z. (2017). Black Phosphorus Quantum Dots with Tunable Memory Properties and Multilevel Resistive Switching Characteristics. Adv. Sci..

[152] Luo M., Cheng W., Zeng X., Mei L., Liu G., Deng W. (2019). Folic Acid-Functionalized Black Phosphorus Quantum Dots for Targeted Chemo-Photothermal Combination Cancer Therapy. Pharmaceutics.

[153] Zhu C., Xu F., Zhang L., Li M., Chen J., Xu S., Huang G., Chen W., Sun L. (2016). Ultrafast Preparation of Black Phosphorus Quantum Dots for Efficient Humidity Sensing. Chem. Eur. J..

[154] Gui R.J., Jin H., Wang Z.H., Li J.H. (2018). Black phosphorus quantum dots: Synthesis, properties, functionalized modification and applications. Chem. Soc. Rev..

[155] Li Y., Li N., Pan W., Yu Z., Yang L., Tang B. (2017). Hollow Mesoporous Silica Nanoparticles with Tunable Structures for Controlled Drug Delivery. ACS Appl. Mater. Interfaces.

[156] Wang J., Pan H., Li J.Y., Nie D., Zhuo Y., Lv Y.S., Wang N., Chen H., Guo S.Y., Gan Y. (2023). Cell membrane-coated mesoporous silica nanorods overcome sequential drug delivery barriers against colorectal cancer. Chin. Chem. Lett..

[157] Takada S., Yamagata Y., Misaki M., Taira K., Kurokawa T. (2003). Sustained release of human growth hormone from microcapsules prepared by a solvent evaporation technique. J. Control. Release.

[158] Saeedi M., Eslamifar M., Khezri K., Dizaj S.M. (2019). Applications of nanotechnology in drug delivery to the central nervous system. Biomed. Pharmacother..

[159] Overchuk M., Weersink R.A., Wilson B.C., Zheng G. (2023). Photodynamic and Photothermal Therapies: Synergy Opportunities for Nanomedicine. ACS Nano.

[160] Choi W., Park B., Choi S., Oh D., Kim J., Kim C. (2023). Recent Advances in Contrast-Enhanced Photoacoustic Imaging: Overcoming the Physical and Practical Challenges. Chem. Rev..

[161] Capozza M., Blasi F., Valbusa G., Oliva P., Cabella C., Buonsanti F., Cordaro A., Pizzuto L., Maiocchi A., Poggi L. (2018). Photoacoustic imaging of integrin-overexpressing tumors using a novel ICG-based contrast agent in mice. Photoacoustics.

[162] Heo M., Jeong J.H., Ju S., Lee S.J., Jeong Y.Y., Lee J.D., Yoo J.W. (2022). Comparison of Clinical Features and Outcomes between SARS-CoV-2 and Non-SARS-CoV-2 Respiratory Viruses Associated Acute Respiratory Distress Syndrome: Retrospective Analysis. J. Clin. Med..

[163] Wei D., Sun Y., Zhu H., Fu Q. (2023). Stimuli-Responsive Polymer-Based Nanosystems for Cancer Theranostics. ACS Nano.

[164] Dunn J.D., Alvarez L.A.J., Zhang X.Z., Soldati T. (2015). Reactive oxygen species and mitochondria: A nexus of cellular homeostasis. Redox Biol..

[165] Yang H., Yao L.H., Wang Y.C., Chen G.J., Chen H. (2023). Advancing cell surface modification in mammalian cells with synthetic molecules. Chem. Sci..

[166] Li Y.J., Wu J.Y., Liu J.H., Xu W.J., Qiu X.H., Huang S., Hu X.B., Xiang D.X. (2021). Artificial exosomes for translational nanomedicine. J. Nanobiotechnol..

[167] Toyofuku M., Schild S., Kaparakis-Liaskos M., Eberl L. (2023). Composition and functions of bacterial membrane vesicles. Nat. Rev. Microbiol..

[168] White J.M., Ward A.E., Odongo L., Tamm L.K. (2023). Viral Membrane Fusion: A Dance Between Proteins and Lipids. Annu. Rev.Virol..

